# Single-Crystalline, Semiconductive Layered Organic Cathode Powers High-Energy All-Solid-State Batteries

**DOI:** 10.1021/acscentsci.6c00267

**Published:** 2026-05-06

**Authors:** Junyong Mo, Jiande Wang, Mircea Dincă

**Affiliations:** † Department of Chemical Engineering, 2167 Massachusetts Institute of Technology, Cambridge, Massachusetts 02139, United States; ‡ Department of Chemistry, Massachusetts Institute of Technology, Cambridge, Massachusetts 02139, United States; § Department of Chemistry, 6740 Princeton University, Princeton, New Jersey 08544, United States

## Abstract

All-solid-state batteries (ASSBs) offer a pathway to improved safety and increased energy density but remain limited by sluggish ion transport and low active material loading in composite cathodes. Organic cathode materials provide a sustainable alternative to metal-based systems, yet their implementation in solid-state architectures is constrained by poor electronic conductivity and inefficient electrode microstructures. Here, we integrate a high-capacity, semiconductive, single-crystalline layered organic cathode into ASSBs and demonstrate an electrochemical performance comparable to that of conventional systems. Systematic optimization of cathode composition identifies a configuration that delivers a specific capacity of 310 mAh g^–1^ at 25 mA g^–1^ with stable cycling over 100 cycles at room temperature under moderate pressure. At this rate, the architecture achieves an active-material-level energy density of 638 Wh kg^–1^. Performance limitations are mitigated through compositing with single-walled carbon nanotubes and operation at an elevated temperature. Electrochemical impedance spectroscopy indicates simplified interfacial behavior and suppressed side reactions relative to conventional solid-state cathodes, while in situ measurements reveal volcano-shaped lithium-ion diffusion behavior arising from the interplay between structural evolution and site occupancy. These results define design constraints for organic solid-state cathodes and establish their viability as functional components in next-generation solid-state energy storage.

## Graphical abstract

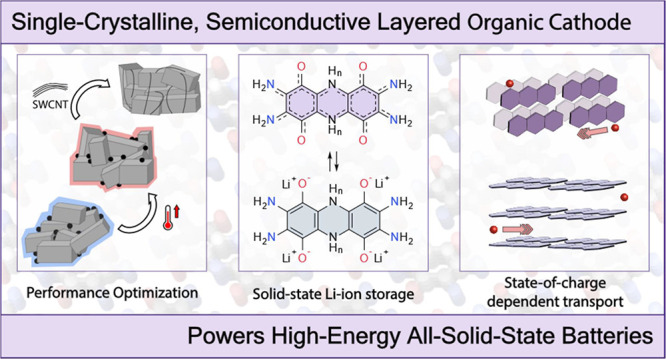

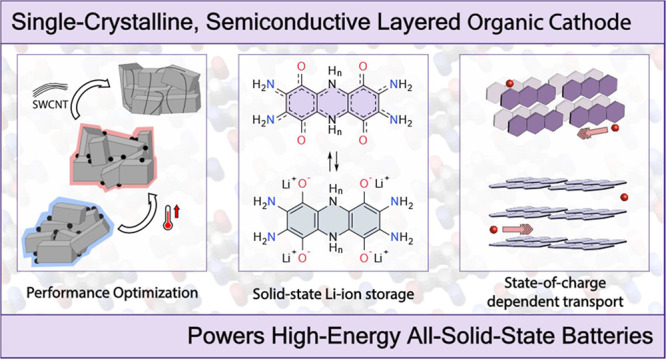

## Introduction

The transition toward electrified transportation and grid-scale energy storage has intensified the demand for battery technologies that exceed the performance limits of conventional lithium-ion systems. All-solid-state batteries (ASSBs) have emerged as a next-generation energy storage platform, offering distinct advantages over liquid electrolyte-based architectures.^1,2^ Replacing flammable organic liquid electrolytes with nonflammable solid electrolytes directly addresses key safety liabilities of conventional batteries, including thermal runaway, electrolyte leakage, and gas evolution.^3,4^ This improved safety profile enables simplified cell and pack designs with reduced cooling and auxiliary safety components, which can translate into higher system-level energy densities.^5,6^

Beyond safety considerations, ASSBs offer intrinsic performance advantages with implications for high-energy applications. The mechanical rigidity of solid electrolytes can, in principle, suppress lithium dendrite propagation on macroscopic length scales, enabling the use of lithium metal anodes with a low redox potential (−3.04 V vs the standard hydrogen electrode) and a theoretical specific capacity exceeding 3,860 mAh g^–1^—nearly an order of magnitude higher than that of graphite.^7^ When paired with high-energy cathode materials, this capability positions ASSBs to surpass the ∼250 Wh kg^–1^ energy densities demonstrated by state-of-the-art liquid electrolyte systems.^8^ In addition, the absence of liquid electrolyte mitigates continuous electrolyte decomposition and repeated solid–electrolyte interphase formation, potentially enabling extended cycle life.^9^ Despite these advantages, widespread deployment of ASSBs remains limited by fundamental challenges, including insufficient ionic conductivity in solid electrolytes, poor electrode–electrolyte interfacial contact, and manufacturing complexity, which must be addressed to fully realize their performance potential.^10–12^

The development of cathode materials remains a primary bottleneck for the practical implementation of ASSBs.^13,14^ Conventional inorganic cathodes, although capable of high energy densities in liquid electrolyte systems, exhibit intrinsic limitations in solid-state architectures. In the absence of liquid-phase wetting, these materials often fail to establish conformal contact with solid electrolytes due to mechanical mismatch, resulting in high interfacial resistance and incomplete utilization of the active material.^15,16^ In addition, many inorganic cathodes undergo substantial volume changes during cycling, which disrupt mechanical contact in rigid solid-state assemblies, while others form chemically incompatible interphases with sulfide electrolytes that progressively degrade performance.^17–19^

Organic electrode materials (OEMs) present an alternative class of cathodes composed of earth-abundant elements such as carbon, nitrogen, and oxygen, offering a pathway toward more sustainable and potentially lower-cost battery chemistries with theoretical specific capacities frequently exceeding 300 mAh g^–1^.^20,21^ Despite these advantages, conventional OEMs suffer from intrinsic limitations that have impeded their deployment, most notably poor electronic conductivity that necessitates high loadings of conductive additives (>30 wt %) and limited structural stability under prolonged cycling.^22,23^ These deficiencies are exacerbated in solid-state configurations, where the absence of liquid electrolyte buffering intensifies transport constraints and amplifies interfacial instability.^24,25^

Here, we show that bis-tetraaminobenzoquinone (TAQ), a layered organic small molecule with distinct structural and electronic characteristics, addresses these limitations and enables robust performance in ASSBs. TAQ crystallizes in a highly ordered two-dimensional layered structure stabilized by extended intermolecular hydrogen-bonding networks, which impart semiconductive behavior with an electronic conductivity exceeding 10^–5^ S cm^–1^.^26,27^ The single-crystalline morphology promotes a uniform electrochemical response and minimizes grain-boundary resistance, a common limitation in polycrystalline cathodes used in solid-state systems. Systematic variation of cathode composition from 20% to 50% active material identifies an optimal formulation that delivers a specific capacity of 310 mAh g^–1^ at 25 mA g^–1^ with stable cycling over 100 cycles at room temperature under moderate pressure (∼5 MPa). Analysis of the rate-dependent performance indicates that mass transport, rather than interfacial charge transfer, constitutes the dominant kinetic limitation. Electrochemical impedance spectroscopy further reveals simplified interfacial behavior and reduced parasitic reactions relative to conventional solid-state cathodes, while in situ measurements uncover volcano-shaped lithium-ion diffusion profiles arising from intrinsic structural evolution and site-occupancy effects. Together, these observations expose transport phenomena that are often masked in liquid electrolyte systems and define design principles for organic cathodes in solid-state energy storage.

## Results and Discussion

### Structure of TAQ and Its Electrochemical Performance in All-Solid-State Configurations

The molecular structure of TAQ is intrinsically compatible with solid-state battery operation and differentiates it from conventional organic electrode materials through several structural attributes. TAQ crystallizes as ordered planar sheets interconnected by intermolecular hydrogen bonding (Figure S1),^26^ giving rise to a highly organized layered architecture. This arrangement supports extended electronic transport pathways that are absent in most organic electrodes, where disordered molecular packing and localized electronic states limit the charge transport. In TAQ, cooperative hydrogen bonding and π–π stacking generate continuous pathways for electron conduction, alleviating a central limitation of organic cathodes in all-solid-state configurations. TAQ undergoes a reversible four-electron redox process (Figure 1a), in which each quinone unit accommodates two lithium ions through sequential reduction steps. This multielectron redox chemistry underpins a theoretical specific capacity of 356 mAh g^–1^, exceeding that of many conventional inorganic cathodes.^28^

**Figure 1:**
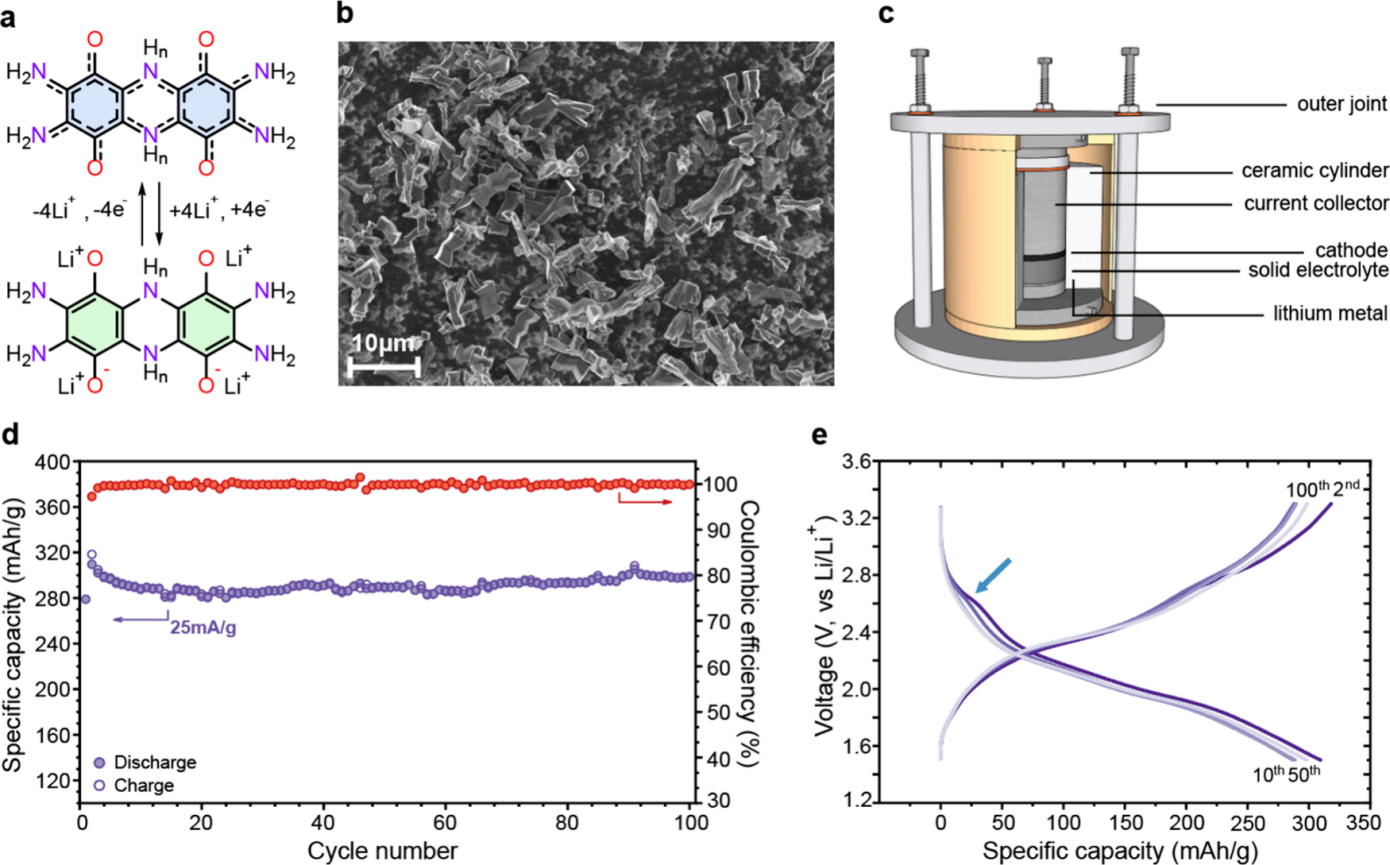
**Molecular and morphological structure of TAQ and its ASSB performance. a**, Molecular structure of TAQ and its four-electron reversible Li-ion storage mechanism (0 ≤ *n* ≤ 1). **b**, SEM image of as-synthesized TAQ single crystals. Scale bar: 10 μm. **c**, Schematic illustration of an optimized all-solid-state battery setup. **d**, Cycling stability test of a 2:7:1 (TAQ:LPSCl:CB) TAQ composite cathode at 25 mA g^–1^ current density. Specific capacity (blue) and Coulombic efficiency (red). **e**, Galvanostatic charge–discharge profiles from selected cycles of a cycling stability test.

The single-crystalline morphology of TAQ, evident from scanning electron microscopy (Figure 1b) and powder X-ray diffraction (Figure S2), further distinguishes it from the polycrystalline materials typically employed in battery electrodes. Single crystallinity promotes spatially uniform electrochemical response and minimizes impedance contributions associated with grain boundaries.^29^ The rod-like crystals exhibit high structural integrity, while the reduced density of grain boundaries suppresses parasitic reactions and mechanical degradation, supporting stable electrochemical behavior during extended cycling under solid-state conditions.

Electrochemical evaluation using the cell configuration shown in Figure 1c demonstrates a robust solid-state performance. Initial electrolyte screening identifies Li_6_PS_5_Cl (LPSCl) as a suitable solid electrolyte for detailed investigation, owing to its superior cycling stability relative to Li_10_GeP_2_S_12_ (LGPS) (Figure S3). A composite cathode with a mass ratio of 2:7:1 TAQ:LPSCl:carbon black (CB) delivers a specific capacity of 310 mAh g^–1^ at 25 mA g^–1^ under moderate stacking pressure (∼5 MPa) at room temperature, corresponding to approximately 87% utilization of the theoretical capacity. Capacity decay is minimal, with 96.4% capacity retention after 100 cycles and a Coulombic efficiency exceeding 99%. Pre- and postcycling powder X-ray diffraction (PXRD) measurements (Figure S4) indicate structural stability and favorable interfacial compatibility within the TAQ–LPSCl composite. The pristine electrode displays characteristic reflections from both LPSCl and TAQ. After 50 cycles, reflections from LPSCl remain prominent, while the TAQ signature peak is attenuated, consistent with reversible interlayer spacing changes during electrochemical cycling. Importantly, no additional reflections attributable to interfacial decomposition products are observed. Scanning electron microscopy of pristine and cycled electrodes (Figure S5) further confirms the preservation of TAQ particle morphology, with no evidence of surface passivation or structural collapse. These observations contrast with the behavior of many inorganic ASSB cathodes, which commonly form resistive interphases with sulfide electrolytes that obscure particle morphology through the accumulation of decomposition layers.^29,30^ Additionally, TAQ’s layered structure enables minimal volumetric changes during cycling (∼6.3%, Figure S6 and Table S1), which maintains interfacial contact and prevents void formation. Together, electrochemical and structural analyses underscore the favorable interfacial compatibility of the TAQ–LPSCl system under solid-state operating conditions.

The galvanostatic charge–discharge (GCD) profiles (Figure 1e) evolve toward sloping voltage characteristics (indicated by the arrow) in contrast to the better-defined plateaus associated with the two sequential two-electron redox processes observed in liquid electrolyte systems.^27,28^ This behavior reflects transport constraints intrinsic to solid-state architectures, where limited electrode–electrolyte contact and constrained ion percolation generate spatial concentration gradients that broaden the effective redox potential distribution. Despite this altered voltage response, the cells exhibit highly reversible cycling with minimal voltage hysteresis and consistent capacity retention across successive cycles, indicating stable electrochemical operation under solid-state conditions. Differential capacity analysis (Figure S7) corroborates this behavior, showing reproducible redox features with stable peak positions over extended cycling, consistent with reversible and well-defined charge-storage processes.

### Optimization of Cathode Composition and Rate Performance Analysis

To optimize cathode composition and active material loading in TAQ-based ASSBs, we systematically evaluated four composite formulations with TAQ:LPSCl:carbon black (CB) mass ratios of 2:7:1, 3:6:1, 4:5:1, and 5:4:1, corresponding to active material loadings of 20%, 30%, 40%, and 50%, respectively. All compositions exhibit stable rate performance over the tested current density range and recover their initial capacities upon returning to lower rates (Figure 2a), indicating reversible electrochemical behavior under varying load conditions. Among the evaluated formulations, the 2:7:1 composition delivers the highest specific capacity across all current densities, retaining 95.6 mAh g^–1^ at 200 mA g^–1^. Increasing the TAQ content from 20% to 40% leads to a systematic decline in specific capacity, consistent with a reduction in the solid electrolyte fraction available to support lithium-ion transport within the composite cathode. At the electrode level, the 4:5:1 composition delivers the highest capacity (91 mAh g^–1^_cathode_) and energy density (231 Wh kg^–1^_cathode_) due to compensatory effects from increased active material loading.

**Figure 2:**
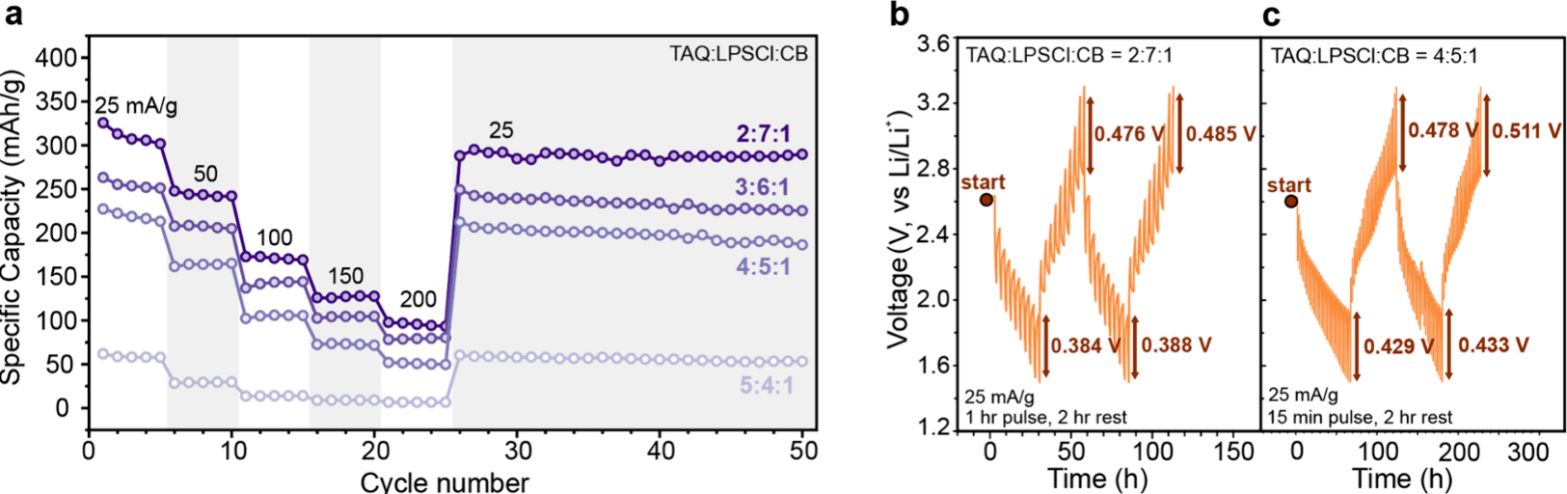
**Rate capability tests for cathode compositions with various TAQ contents and GITT data. a**, Rate capability tests at six different charge/discharge rates of four different cathode composition ratios. **b**, Galvanostatic intermittent titration technique (GITT) data for a 2:7:1 TAQ cathode. Charge/discharge rate: 25 mA g^–1^, 1 h pulse, 2 h rest. **c**, GITT data for a 4:5:1 TAQ cathode. Charge/discharge rate: 25 mA g^–1^, 15 min pulse, 2 h rest.

Performance degradation becomes pronounced as the TAQ content increases from 40% to 50%, with the 5:4:1 composition exhibiting substantially reduced capacity and poor rate retention relative to the 4:5:1 formulation. This sharp decline is consistent with a reduction of the solid electrolyte fraction below the percolation threshold required for continuous ion transport.^31,32^ At 50% active material loading, the electrolyte content (40%) approaches the minimum necessary to sustain effective lithium-ion conduction pathways, leading to increased transport tortuosity and elevated ionic resistance. This conclusion is supported by maintaining a constant carbon black fraction of 10% across all compositions to ensure sufficient electronic conductivity, thus isolating ion transport as the dominant performance-limiting factor at higher TAQ loadings. We believe that advanced manufacturing techniques, such as solvent-assisted processing and high-energy ball milling, can address the reduced capacities observed at higher mass loadings in TAQ composite cathodes since all of the TAQ–LPSCl–CB composite cathodes investigated in this work were prepared by manual hand-grinding.

To further probe transport limitations across different cathode compositions, galvanostatic intermittent titration technique (GITT) measurements were performed on representative 2:7:1 and 4:5:1 cathode formulations. The resulting GITT profiles (Figures 2b and 2c) show a systematic increase in overpotential with a higher TAQ loading. Specifically, the 2:7:1 composition exhibits overpotentials of 0.384 V/0.388 V and 0.476 V/0.485 V at the first fully discharged and charged states, respectively, whereas the 4:5:1 composition displays higher corresponding values of 0.429 V/0.433 V and 0.478 V/0.511 V. The elevated overpotentials observed at higher TAQ content are consistent with increased mass transport resistance arising from reduced solid electrolyte fraction within the composite cathode. These results support the conclusion that ion transport, rather than interfacial charge transfer, constitutes the rate-determining process in TAQ-based ASSBs.

### Impact of TAQ Crystallinity on All-Solid-State Battery Performance

The electrochemical performance of TAQ in all-solid-state configurations is strongly dependent on its crystallinity. Control over TAQ crystallinity was achieved during synthesis by using salt additives that modulate precursor and product solubility.^26^ High-crystallinity TAQ was synthesized in-house from tetraaminobenzoquinone (TABQ) using tetrabutylammonium chloride (TBACl), whereas low-crystallinity material was obtained from commercial TABQ using tetrabutylammonium bromide (TBABr) without further purification. Scanning electron microscopy revealed pronounced morphological differences between the two materials. Low-crystallinity TAQ consists of nanocrystalline particles (∼200 nm) with poorly defined facets (Figure S10), in contrast to the well-faceted microcrystals observed for high-crystallinity TAQ (Figure 1b). PXRD further corroborates this distinction (Figure S11): although both samples exhibit identical peak positions, confirming the same crystal polymorph, the low-crystallinity material shows broadened and attenuated reflections indicative of a reduced crystallite domain size. These differences in crystallinity translate directly into substantial performance disparities in ASSBs. High-crystallinity TAQ delivers first-charge capacities of 263 mAh g^–1^ and 227 mAh g^–1^ for the 3:6:1 and 4:5:1 cathode formulations, respectively, whereas the corresponding low-crystallinity samples achieve only 215 mAh g^–1^ and 78 mAh g^–1^, representing capacity reductions of 18% and 66% (Figure S12). In addition to reduced capacity, cathodes fabricated with low-crystallinity TAQ exhibit premature cell failure, further underscoring the critical role of crystallinity in enabling stable and efficient solid-state electrochemical performance (Figure S13).

The heightened importance of single crystallinity in all-solid-state configurations arises from the transport and interfacial constraints intrinsic to solid-state battery architectures. Reduced grain boundary density minimizes high-resistance pathways for both electronic and ionic conduction, an effect that is especially consequential in solid-state systems where transport limitations are more severe than in liquid electrolyte counterparts.^14,33^ Single-crystalline particles also exhibit greater mechanical robustness, mitigating fracture and loss of interparticle contact during cycling, while their uniform lattice structure reduces surface heterogeneity and suppresses the formation of chemically unstable interfacial species.^34^ Collectively, these factors account for the disproportionately large performance penalties associated with reduced crystallinity in ASSBs and establish crystal quality as a central design parameter for organic electrode materials intended for solid-state energy storage.

### Performance Enhancement Strategies for High Active Material Loading

To further improve the performance of TAQ-based ASSBs, particularly at highly active material loadings, two complementary strategies were explored. One approach employs a TAQ–single-walled carbon nanotube (SWCNT) composite formed via in situ synthesis in which carboxyl-functionalized SWCNTs are incorporated into TAQ crystals during TABQ dimerization. This process generates one-dimensional line contacts between TAQ and the conductive additive, in contrast to the zero-dimensional point contacts formed with conventional carbon black (CB) (Figure 3a). The resulting composite preserves the crystalline structure of TAQ (Figure S15) while establishing continuous electronic pathways, enabling electrode formulations with a reduced CB content and increased solid electrolyte fraction. Scanning and transmission electron microscopy (Figure 3b,c) confirm the successful integration of SWCNTs within the TAQ crystal matrix, with nanotubes observed to wrap around and penetrate individual TAQ crystals. This intimate interfacial contact facilitates efficient electronic coupling while preserving the single-crystalline morphology that is critical for solid-state electrochemical performance. The enhanced electronic conductivity provided by the SWCNT network allows for cathode formulations with lower CB content, creating additional compositional flexibility to increase the solid electrolyte loading without sacrificing the active material fraction. Electrochemical evaluation of a 50:45:3.2:1.8 TAQ:LPSCl:CB:SWCNT composite, with the TAQ-to-SWCNT ratio verified by elemental analysis (Table S2), demonstrates the effectiveness of this strategy relative to the baseline 5:4:1 formulation without SWCNTs (Figure 3d). The TAQ–SWCNT composite cathode delivers a specific capacity of 139 mAh g^–1^ at 25 mA g^–1^ and exhibits improved rate retention, particularly at higher current densities, underscoring the role of engineered electronic percolation in mitigating transport limitations at elevated active material loadings.

**Figure 3:**
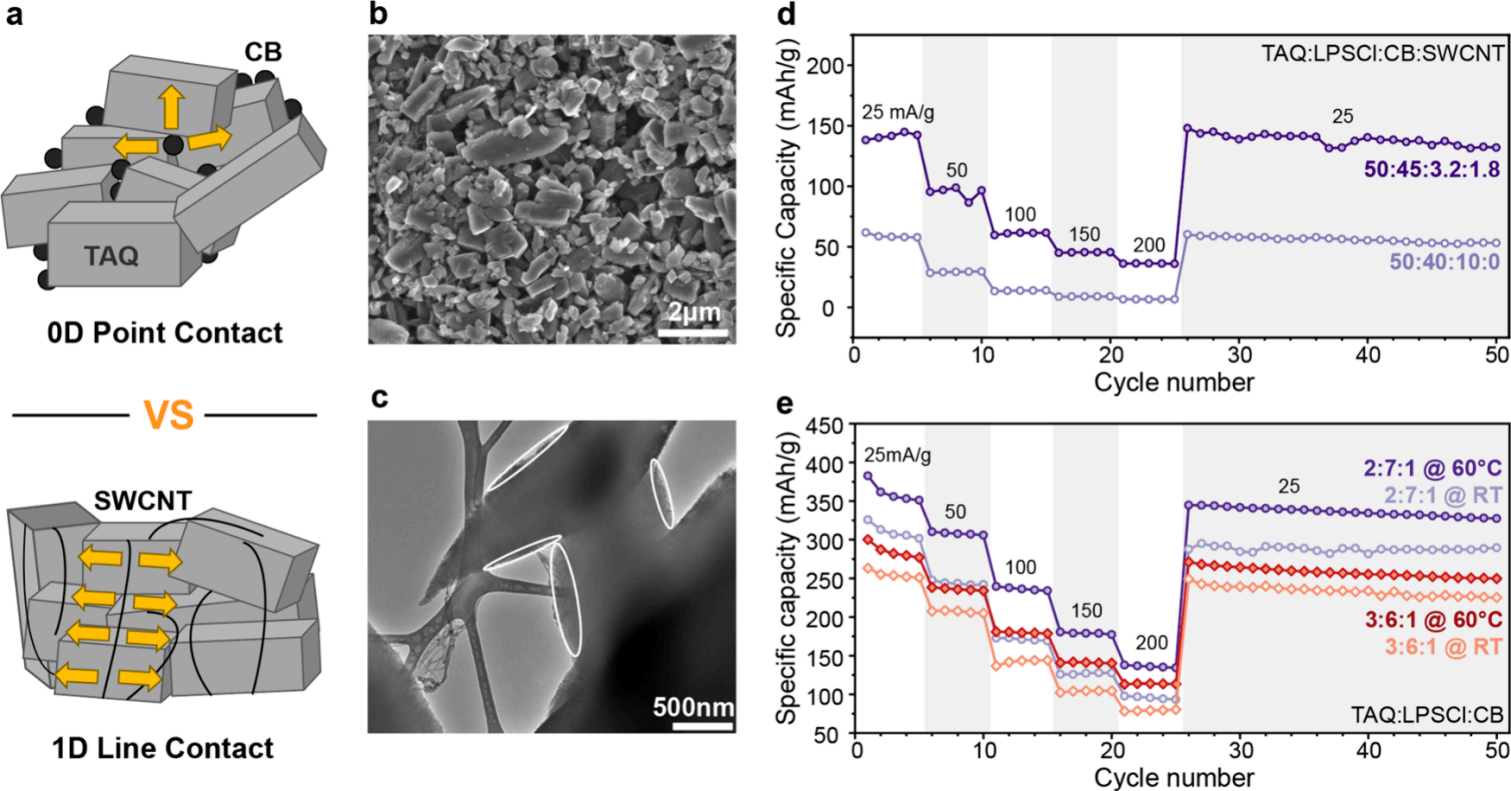
**Performance enhancement strategies for higher TAQ content cathodes. a**, Schematic illustration comparing zero-dimensional point contacts and one-dimensional line contacts using neat TAQ with CB and SWCNT-TAQ composite, respectively. **b**, SEM and **c**, TEM images of as-synthesized SWCNT-TAQ composite. Scale bars: 2 μm (b), 500 nm (c). White solid lines indicate SWCNTs wrapping around the TAQ particles. **d**, Rate capability test of an SWCNT-TAQ composite cathode, maintaining TAQ content at the 50 wt % level. **e**, Rate capability test of two different cathode compositions (2:7:1 and 3:6:1) at elevated temperature (60 °C).

A second performance-enhancement strategy involves operation at elevated temperature to leverage the thermally activated, hopping-based ionic conduction of the solid electrolyte.^35^ Rate capability measurements of both the 2:7:1 and 3:6:1 cathode compositions at 60 °C show marked improvements relative to room-temperature operation (Figure 3e). Under these conditions, the 2:7:1 formulation achieves a specific capacity of approximately 353 mAh g^–1^ at 25 mA g^–1^, corresponding to near-complete utilization of TAQ’s theoretical capacity (356 mAh g^–1^). The substantial capacity increase observed at elevated temperature indicates that the limitations encountered under ambient conditions arise predominantly from mass transport constraints within the solid electrolyte rather than intrinsic redox limitations of TAQ. The 3:6:1 composition exhibits a similar trend, with enhanced capacity and rate performance at 60 °C approaching that of the optimized 2:7:1 formulation operated at room temperature. Together, these results demonstrate that elevated-temperature operation can partially offset reduced solid electrolyte content, enabling higher active material loadings while maintaining robust electrochemical performance and establishing temperature modulation as an effective strategy for mitigating transport limitations in TAQ-based ASSBs.

### Electrochemical Impedance Analysis and Lithium-Ion Diffusion Kinetics

To obtain quantitative insight into the transport limitations governing TAQ-based ASSB performance, electrochemical impedance spectroscopy (EIS) was conducted across multiple cathode compositions. Ex situ EIS measurements collected during early cycling were performed in the fully discharged state for the 2:7:1, 3:6:1, and 5:4:1 formulations. The resulting Nyquist plots display impedance responses that differ markedly from those typical of liquid electrolyte lithium-ion batteries, exhibiting predominantly linear behavior (Figure 4a) rather than distinct semicircular features followed by Warburg diffusion tails.^36^ This linear impedance response can be described using a simplified equivalent circuit model that reflects the interfacial characteristics of the TAQ–solid electrolyte system during initial cycling (Figure S16). In conventional lithium-ion batteries, impedance spectra are commonly interpreted using a Randles-type circuit comprising bulk electrolyte resistance (R_bulk_), charge-transfer resistance (R_ct_), cathode–electrolyte interphase resistance (R_CEI_) with associated constant-phase elements, and a Warburg element representing semi-infinite diffusion.^36,37^ In contrast, the absence of pronounced semicircular features in the TAQ-based solid-state cells indicates that interphase-related resistive and capacitive elements contribute minimally, relative to the dominant transport processes.

**Figure 4:**
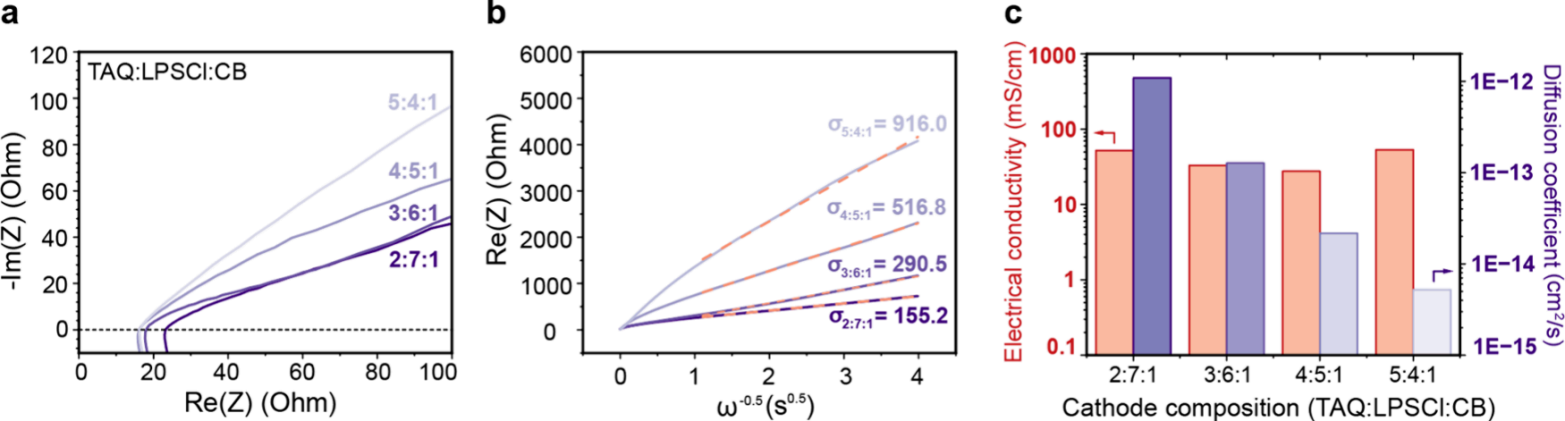
**Ex situ electrochemical impedance spectroscopy and lithium-ion diffusion coefficients at the fully discharged state for different cathode composition ratios. a**, Nyquist plots of the four different cathode compositions. **b**, ω^–0.5^ vs Re­(Z) plots (solid lines), linear regression fits at the low frequency regime (dashed lines), and their slope values (Warburg coefficients). **c**, Electrical conductivities (left axis) and calculated lithium-ion diffusion coefficient values (right axis).

This behavior is consistent with two features of the TAQ–solid electrolyte interface. First, all-solid-state configurations inherently limit interfacial reactivity by eliminating easily decomposable lithium salts and carbonate solvents, thereby suppressing cathode–electrolyte interphase formation.^38^ Second, the purely organic nature of TAQ, which lacks transition-metal centers capable of catalyzing interfacial decomposition reactions, further reduces the likelihood of chemically resistive interphase growth compared to conventional inorganic cathodes.^39^ In contrast, many inorganic cathode materials in solid-state systems form chemically incompatible interphases with sulfide electrolytes that introduce substantial additional impedance.^40,41^ Accordingly, the impedance response of the TAQ-based cells is adequately captured by a reduced circuit consisting primarily of R_bulk_, R_ct_, and a diffusion-associated impedance element, yielding linear Nyquist plots with x-intercepts corresponding to the combined R_bulk_ and R_ct_ contributions. The systematic increase in x-intercept values with increasing solid electrolyte fraction (Figure 4a) supports this assignment, as LPSCl is electronically insulating, while TAQ exhibits semiconductive behavior, leading to composition-dependent variations in effective charge-transfer resistance.

Quantitative lithium-ion diffusion coefficients were extracted from the impedance data using their relationship to the Warburg impedance (Supplementary Note). Linear regression of the Warburg region (Figure 4b) yields well-defined linear behavior from early cycle EIS measurements, enabling the reliable extraction of the Warburg coefficients. The resulting slopes (σ_2:7:1_ = 155.2, σ_3:6:1_ = 290.5, σ_4:5:1_ = 516.8, σ_5:4:1_ = 916.0) exhibit systematic variation across compositions, reflecting composition-dependent mass transport characteristics that were subsequently used to calculate lithium-ion diffusion coefficients. Comparison of the electronic conductivity and ionic diffusion behavior (Figure 4c) reveals a clear decoupling between these two transport processes. The electronic conductivity, measured by a two-probe method (Figure S17), remains relatively invariant across all compositions, ranging from 25 to 50 mS cm^–1^. In contrast, the lithium-ion diffusion coefficients span several orders of magnitude, decreasing from approximately 10^–12^ to 10^–15^ cm^2^ s^–1^ as the solid electrolyte fraction decreases. The diffusion coefficient exhibits a clear inverse correlation with TAQ content, declining systematically as the solid electrolyte loading is reduced from 70% to 40%.

These results quantitatively confirm that ionic mass transport rather than electronic conductivity governs performance limitations in TAQ-based ASSBs. Distribution of relaxation times (DRT) analysis (Figure S18) supports this conclusion, showing dominant peaks at longer time scales characteristic of diffusive mass-transfer processes. Collectively, these observations provide a mechanistic basis for the strong composition-dependent trends observed in the electrochemical performance.

### Lithium-Ion Transport as a Function of the State of Charge (SOC)

In situ EIS reveals the evolution of lithium-ion diffusion kinetics as a function of the state of charge (SOC) across different cathode compositions. EIS measurements were collected at discrete voltage points during a single discharge–charge cycle (Figure 5a), allowing diffusion coefficients to be extracted from the low-frequency Warburg response over the full operating voltage window (Figures S19–S22). Across all four cathode compositions, the resulting diffusion coefficients exhibit pronounced volcano-shaped dependences on SOC (Figure 5b–e). This nonmonotonic behavior reflects the coupled influence of site occupancy and structural evolution on lithium-ion transport. Under Li-poor conditions at high voltage, contraction of the TAQ interlayer spacing restricts ion mobility and suppresses diffusion kinetics. Conversely, under Li-rich conditions at low voltage, the limited availability of vacant sites constrains hopping-mediated transport pathways despite expanded lattice spacing (Figure 6).^42^ The diffusion coefficient reaches a maximum at intermediate SOC, corresponding to an optimal balance between structural accessibility and site availability. This behavior is conceptually consistent with a θ­(1 – θ) dependence, where θ denotes the fractional lithium occupancy.

**Figure 5:**
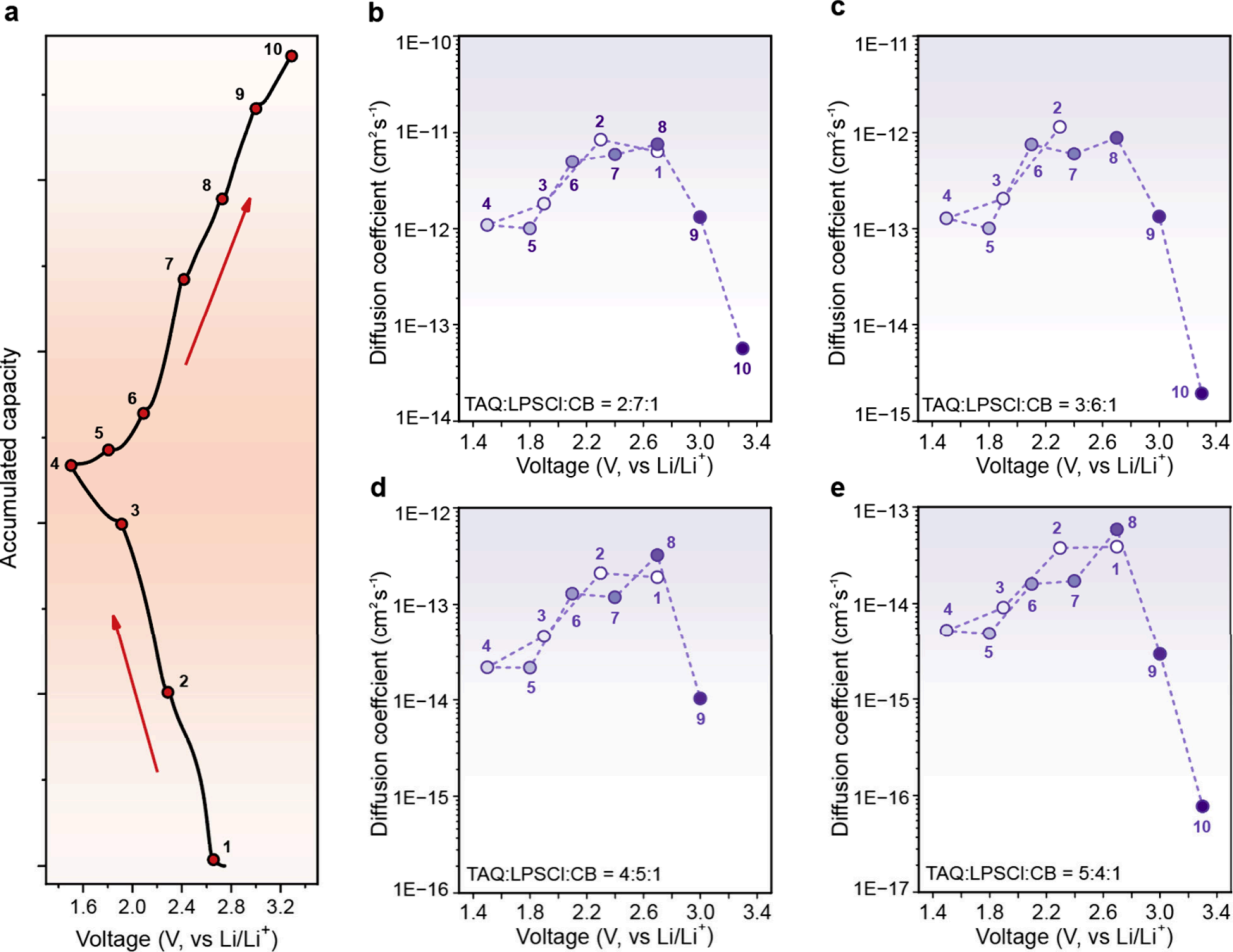
**In situ electrochemical impedance spectroscopy and SOC-dependent Li-ion diffusion coefficients. a**, Representative voltage profile indicating the ten voltage points for in situ EIS measurements. **b, c, d, e**, SOC-dependent Li-ion diffusion coefficients for 2:7:1, 3:6:1, 4:5:1, and 5:4:1 (TAQ:LPSCl:CB) cathodes, respectively.

**Figure 6:**
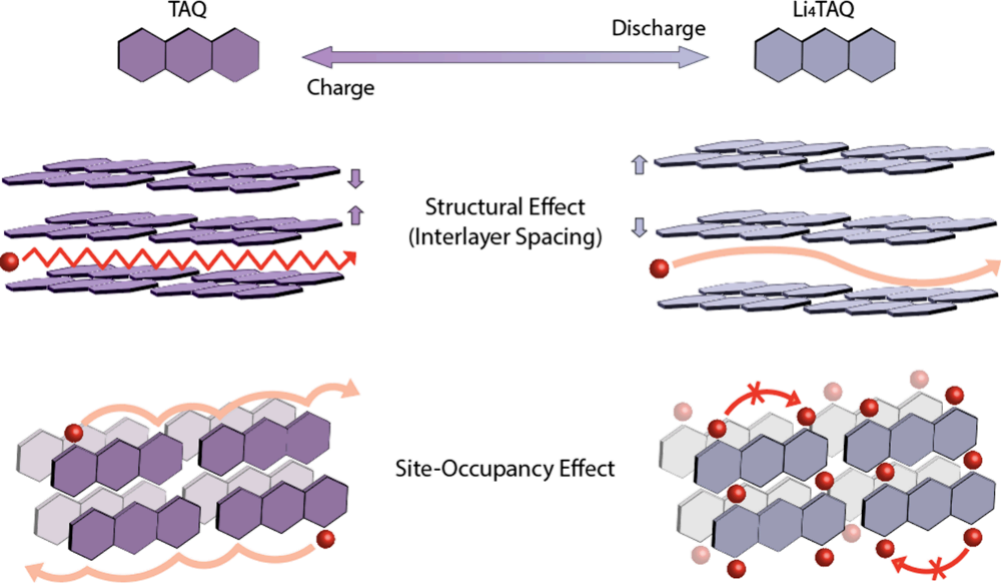
**Schematic illustration of structural and site-occupancy effects governing lithium-ion diffusion in TAQ ASSBs.** Expanded interlayer spacing in Li_4_TAQ enables higher lithium-ion mobility compared with TAQ through the structural effect. Abundant accessible vacant hopping sites in TAQ lead to facilitated lithium-ion diffusion compared with Li_4_TAQ through the site-occupancy effect.

The SOC-dependent diffusion behavior is closely linked to the structural evolution of TAQ’s layered framework. TAQ undergoes reversible interlayer expansion upon lithiation,^28^ which increases the effective diffusion channel size and lowers migration barriers at intermediate lithium contents, whereas the fully delithiated state exhibits contracted interlayer spacing that constrains ion transport. This structural responsiveness contributes directly to the pronounced volcano-shaped diffusion profiles observed in the TAQ-based ASSBs. Such behavior contrasts with that of conventional batteries, in which liquid electrolytes provide continuous ionic pathways that buffer local carrier limitations and reduce sensitivity to host lattice changes.^43,44^ Many inorganic cathode materials either undergo discrete phase transitions or retain comparatively rigid crystal structures, resulting in more uniform Li-ion transport across the state-of-charge window.^45–47^ Consequently, SOC-dependent modulation of the binding environment and transport landscape is typically less pronounced in these systems.

Notably, volcano-shaped diffusion behavior has been reported in a limited number of intercalation-type inorganic cathodes, where it has been attributed to variations in vacancy concentration and contraction of the crystallographic *c* parameter during deintercalation.^48^ This precedent is consistent with the present observations, as TAQ’s layered structure supports rapid two-dimensional cation transport^27^ and exhibits intercalation–like kinetic characteristics that couple lattice evolution and site occupancy to Li-ion diffusion.

The hopping-mediated diffusion mechanism in TAQ, reflected in the (1 – θ) dependence on vacant site availability, can be rationalized by features of its molecular structure. In contrast to many organic electrode materials with relatively homogeneous charge distributions, TAQ contains multiple redox-active and polar functional groups, including quinone moieties and free amine groups, which generate a heterogeneous electrostatic potential landscape.^26^ This heterogeneity gives rise to distinct metastable lithium binding sites associated with specific coordination environments, favoring site-to-site hopping as the dominant transport mechanism rather than continuous band-like diffusion. Across all compositions, the lithium-ion diffusion coefficient at the fully charged state (3.3 V vs Li/Li^+^) is markedly lower than that at intermediate states of charge. This pronounced suppression is consistent with reduced vacant-site availability but may also reflect additional interfacial contributions. Specifically, operation at potentials approaching or exceeding the electrochemical stability window of LPSCl^49,50^ may induce minor interfacial degradation and the formation of resistive surface layers, further impeding ion transport beyond intrinsic structural and site-occupancy effects.

## Conclusion

This work establishes bis-tetraaminobenzoquinone (TAQ) as a high-performing organic cathode material for all-solid-state batteries, delivering a specific capacity of 310 mAh g^–1^ at room temperature under moderate external pressure with stable cycling over 100 cycles. The observed performance arises from the combination of single-crystalline morphology, a two-dimensional layered framework, and intrinsic semiconductive behavior, which together support effective charge transport under solid-state operating conditions. Systematic optimization of cathode composition reveals that ionic mass transport rather than interfacial charge transfer or electronic conductivity constitutes the dominant performance constraint, with continuous solid electrolyte percolation becoming critical at electrolyte loadings below 50%.

EIS analysis provides quantitative evidence that TAQ-based ASSBs exhibit simplified interfacial behavior during early cycling, consistent with the reduced interfacial reactivity expected for organic cathodes in solid-state configurations. Most notably, lithium-ion transport in these systems is governed by coupled site-occupancy and structural effects that manifest as pronounced volcano-shaped diffusion profiles as a function of the state of charge. This behavior, described by a θ­(1 – θ) dependence, contrasts with the comparatively uniform diffusion characteristics typical of conventional liquid electrolyte systems and highlights transport phenomena that are uniquely accessible in organic solid-state electrodes.

Targeted performance-enhancement strategies further demonstrate the tunability of this system. Incorporation of single-walled carbon nanotubes establishes continuous electronic pathways that mitigate percolation limitations at high active material loadings, while elevated-temperature operation leverages thermally activated ionic conduction in the solid electrolyte. Under these conditions, TAQ-based ASSBs achieve a specific capacity of 353 mAh g^–1^ at 60 °C, corresponding to 99.2% utilization of the theoretical capacity, without compromising structural integrity or redox reversibility.

Together, these results define key design principles for organic cathodes in all-solid-state batteries, emphasizing the central roles of crystallinity, lattice adaptability, and site-resolved transport. Comparative analysis against state-of-the-art cathodes in LPSCl-based systems (Table S3) confirms TAQ’s competitive performance and validates these design principles. By elucidating transport mechanisms that are often masked in liquid electrolyte systems, this work demonstrates that organic electrode materials can be engineered to operate efficiently in solid-state architectures and identifies new opportunities for safe, sustainable, and high-performance energy storage based on molecularly designed solids.

## Supplementary Material



## Notes

Additional experimental procedures, materials, methods, characterization data, and supplementary analyses (PDF)
