# Supplementary material for: Single-Crystalline, Semiconductive Layered Organic Cathode Powers High-Energy All-Solid-State Batteries
Source: ACS Cent Sci. 2026 May 6;12(5):695–703. doi: 10.1021/acscentsci.6c00267 (PMC13220202; doi:10.1021/acscentsci.6c00267)
Supplement: Supplementary file 1 [file oc6c00267_si_001.pdf]

Supporting Information for

Single-Crystalline, Semiconductive Layered Organic Cathode Powers High-Energy All-Solid-State Batteries

Junyong Mo,[1†] Jiande Wang,[2†] Mircea Dincă[3*]

[1]Department of Chemical Engineering, Massachusetts Institute of Technology, Cambridge, MA 02139, United States

[2]Department of Chemistry, Massachusetts Institute of Technology, Cambridge, MA 02139, United States

[3]Department of Chemistry, Princeton University, Princeton, NJ 08544, United States

[†]These authors contributed equally to this work

*Correspondence: mdinca@princeton.edu

Table of Contents

Materials, characterization, synthetic, and electrochemical methods ------------------------------------- S2

Supporting note -- S4

Supporting figures and tables --- S5

References -- S30

Materials, characterization, synthetic, and electrochemical methods

Materials

All commercially available chemicals were used without further purification unless otherwise noted. Tetraaminobenzoquinone was synthesized following a previous literature report.[1] Carboxylic acid functionalized single-walled carbon nanotubes (>90% carbon basis, D × L 4-5nm × 0.5-1.5 μm) and lithium phosphorus sulfur chloride (LPSCl, argyrodite solid electrolyte, >99.5 %, powder, battery grade) were purchased from Sigma-Aldrich. Lithium germanium phosphorus sulfide (>99.9 %, coarse powder) was purchased from MSE Supplies.

Characterization methods

Powder X-ray diffraction (PXRD): PXRD patterns were obtained using a Bruker D8 diffractometer with a 2θ reflection geometry and Cu Kα radiation. Measurements were conducted at 40 kV and 40 mA. Samples were prepared by depositing a thin layer of powder sample on a zero-background silicon substrate. For air-sensitive materials, an X-ray transparent airtight specimen holder was employed during analysis.

Elemental analyses: Elemental analyses were performed by Robertson Microlit Laboratories, Ledgewood, NJ, United States.

Scanning electron microscopy (SEM): SEM images were acquired using a Zeiss Merlin high-resolution scanning electron microscope equipped with an InLens detector at MIT.nano. Images were obtained at an operating voltage of 3-4 kV.

High-resolution cryogenic transmission electron microscopy (cryo-HRTEM): High-resolution cryo-EM images were acquired using a Talos Arctica G2 microscope at MIT.nano.

Electrical conductivity measurements: Electrical conductivity measurements were conducted at room temperature under ambient atmosphere. Two-probe measurement technique was applied on a pressed pellet using ASSB setup.[2]

Synthetic methods

Bis-tetraaminobenzoquinone (TAQ): Tetraaminobenzoquinone (TABQ), the precursor to TAQ, was prepared following a solvothermal method reported previously.[1] To summarize, for the high-crystallinity TAQ, 284 mg (1.69 mmol, 1 equiv.) of house-synthesized TABQ and 880 mg (3.17 mmol, 1.88 equiv.) of anhydrous tetrabutylammonium chloride (TBACl) were added to a 250 mL pressure vessel charged with 60 mL of N,N-dimethylformamide (DMF). The reaction mixture was heated at 120°C for 12 hr, cooled down to room temperature, filtered, and washed with fresh DMF until the color of the filtrate became colorless. For the low-crystallinity TAQ, 284 mg (1.69 mmol, 1 equiv.) of commercial TABQ and 1.021 g (3.17 mmol, 1.88 equiv.) of anhydrous tetrabutylammonium bromide (TBABr) were added to a 250 mL pressure vessel charged with 60 mL of DMF. The reaction mixture was heated at 120°C for 12 hr, cooled down to room temperature, filtered, and washed with fresh DMF until the color of the filtrate became colorless. Collected products were further washed with ethanol and methanol then dried at 70°C under reduced pressure.

TAQ-SWCNT composite: TAQ-SWCNT composite was prepared following a solvothermal method reported previously. To summarize, 1 mg of carboxylic acid functionalized SWCNT was uniformly dispersed in 40 mL of DMF in a 250 mL pressure vessel, then sonicated for 2 hr. 100 mg (0.595 mmol, 1 equiv.) of house-synthesized TABQ and 830 mg (2.99 mmol, 5.02 equiv.) of anhydrous TBACl were

added to the pressure vessel. The reaction mixture was sonicated for 30 min, heated at 120°C for 12 hr, cooled down to room temperature, filtered, and washed with fresh DMF until the color of the filtrate became colorless. Collected products were further washed with ethanol and methanol then dried at 70°C under reduced pressure.

Electrochemical methods

Electrode fabrication: Every cathode composition with neat TAQ and CB was prepared on a total 50mg basis. A specific amount of TAQ and CB were added to a mortar and hand-ground for 20 min. The ground powder mixture was then collected and its weight was measured. It was then transferred into an argon-filled glovebox, and a specific amount of Li_6PS_5Cl powder was added based on the relative ratio. The mixture was ground once again for 20 min with a mortar and a pestle. The final powder mixture was transferred to a 20mL glass scintillation vial and stored under argon at room temperature.

Assembly of all-solid-state cells: First, 100 mg of the Li_6PS_5Cl powder was cold pressed in a cylinder with a diameter of 10 mm under 100 MPa to fabricate a solid electrolyte (SE) pellet. Next, the composite cathode powder was evenly spread onto one side of the SE pellet and cold pressed under 500 MPa for 1 min. The active material loadings were maintained between 2-3 mg/ cm^2. Finally, a thin lithium foil was pressed onto the other side of the SE pellet. Complete ASSB batteries were assembled and subjected to external pressure of ~5 MPa for further experiments.

Cell cycling and rate capability tests: Battery cycling and rate capability tests were conducted using a Landt battery cycler. Cycling stability and rate capability were tested at room temperature unless otherwise noted.

Galvanostatic intermittent titration technique (GITT): GITT measurements were carried out using a Landt battery cycler. A current density of 25 mA g^{-1} was applied unless otherwise noted. Cutoff voltages of 1.5 V and 3.3 V were applied. Measurements were conducted for at least six discharge-charge cycles.

Electrochemical impedance spectroscopy (EIS): EIS analyses were performed using a BioLogic SP-200 potentiostat at frequencies between 1 MHz and 10 mHz.

Supporting Note

Diffusion coefficients were estimated using previously reported equations.[3,4] Assuming semi-infinite diffusion,

$$D = 0.5\left(\frac{RT}{AF^2\sigma C}\right)^2$$

where the Warburg coefficient σ can be calculated by a relationship:

$$Z_{re} = R_{equiv} + \sigma\omega^{-0.5}$$

in the low frequency regime. Here, D is the lithium-ion diffusion coefficient (cm^2/s), R is the gas constant (8.314 J mol^{-1} K^{-1}), T is the temperature (298.15 K for room temperature experiments), A is the electrode surface area (cm^2), F is the Faraday constant (96485 C/mol), and C is the lithium-ion molar concentration (mol/cm^3).

Supporting figures and tables

a

b

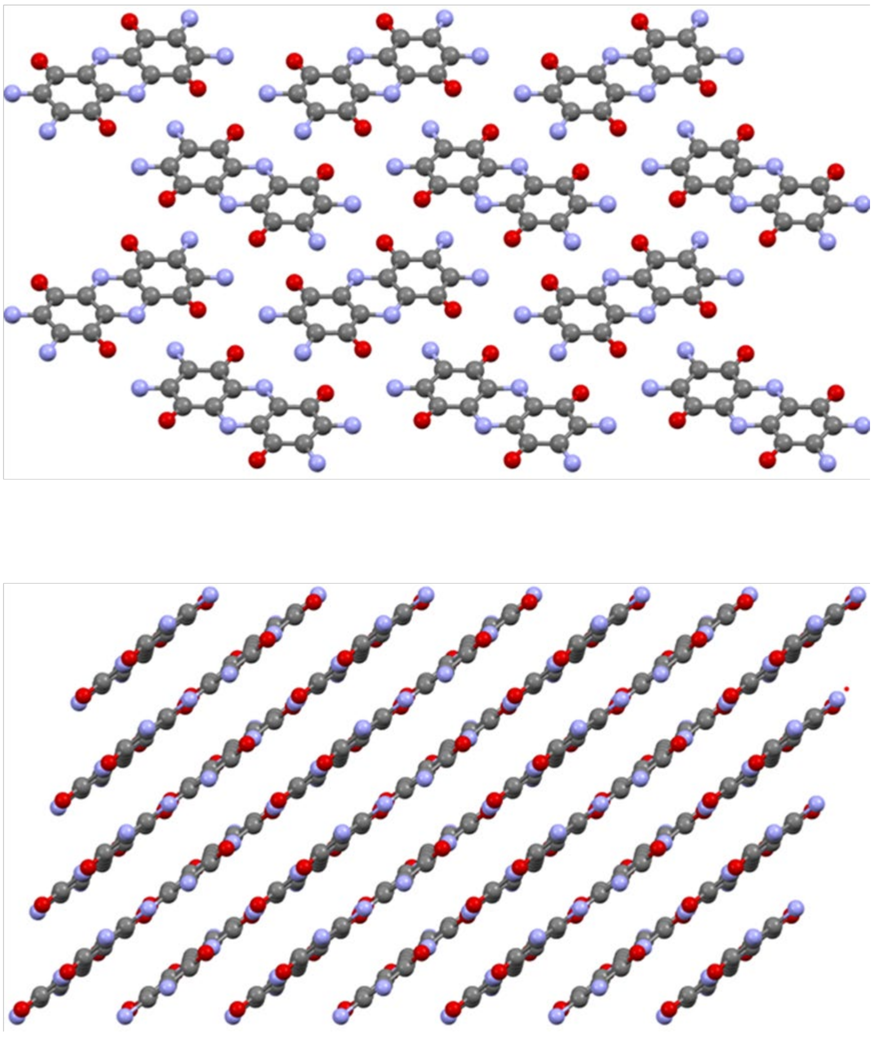

Figure S1. Ball-and-stick representation of TAQ molecular structure. **a,** In-plane molecular packing viewed along the a-axis, showing close intermolecular distances that facilitate extensive hydrogen bonding networks. **b,** Out-of-plane molecular packing viewed along the b-axis, revealing the two-dimensional layered structure with an interlayer distance of 3.14 Å.

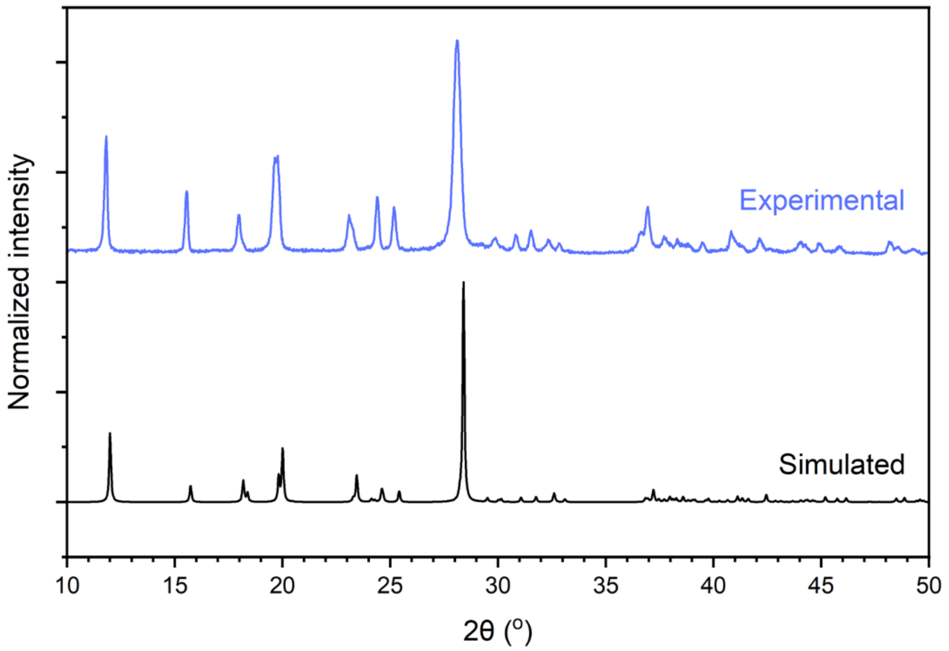

Figure S2. PXRD patterns of experimental (synthesized) and simulated TAQ.

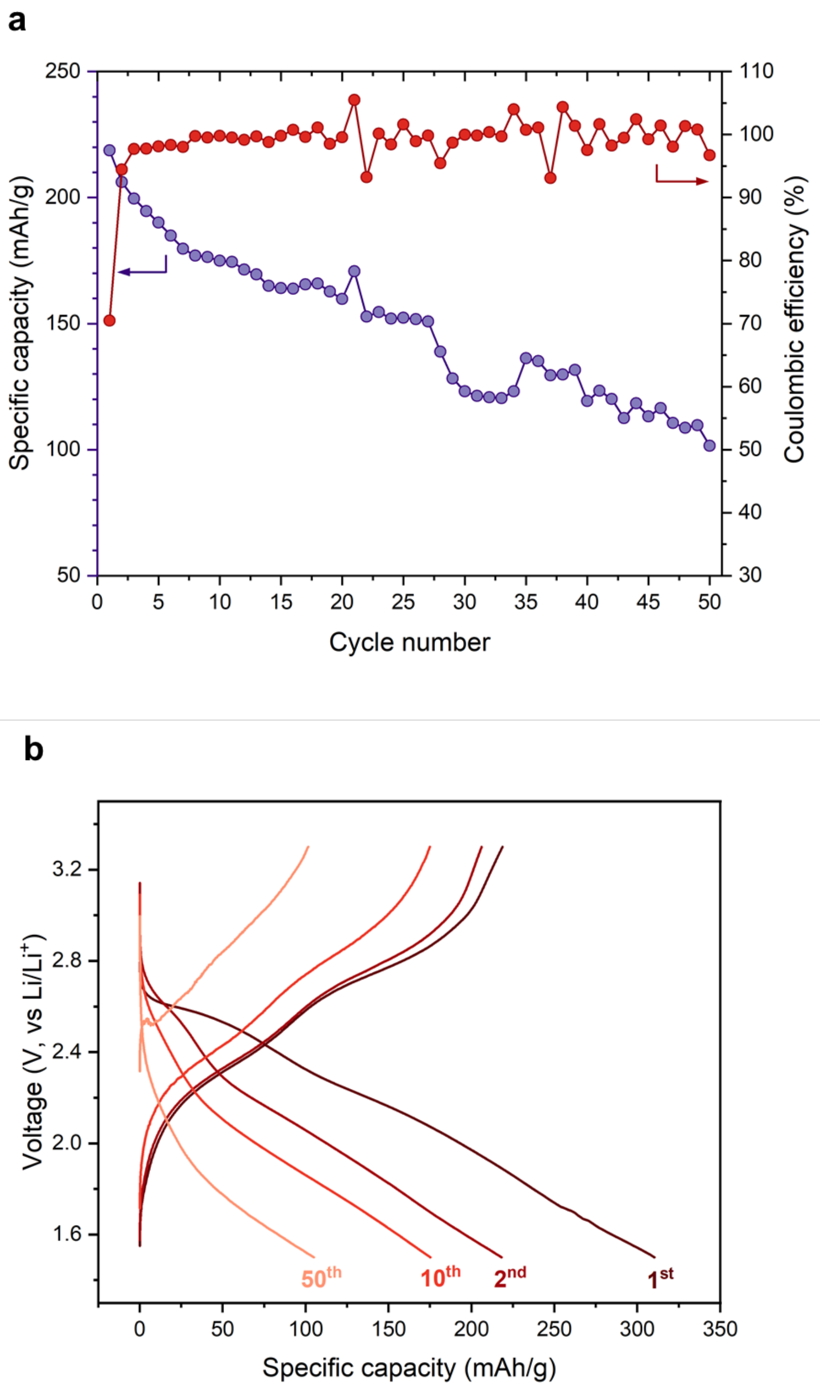

Figure S3. Cycling performance and galvanostatic charge-discharge (GCD) profiles at selected cycles for the 2:7:1 (TAQ:LGPS:CB) cathode cycled at 25 mA g^{-1}.

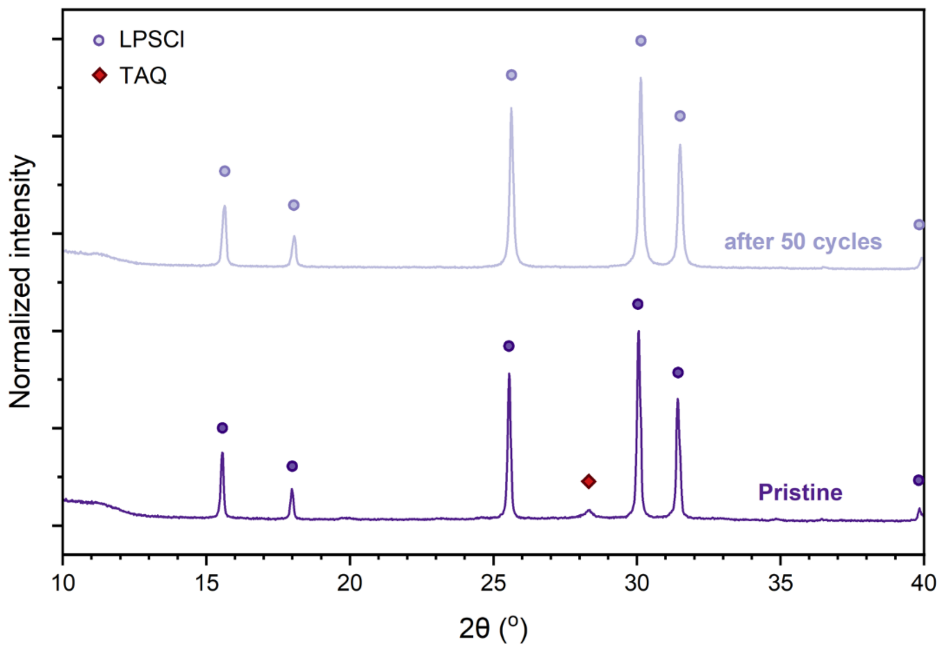

Figure S4. PXRD patterns of 2:7:1 (TAQ:LPSCl:CB) cathode before cycling (pristine) and after 50 cycles at 25 mA g^{-1}.

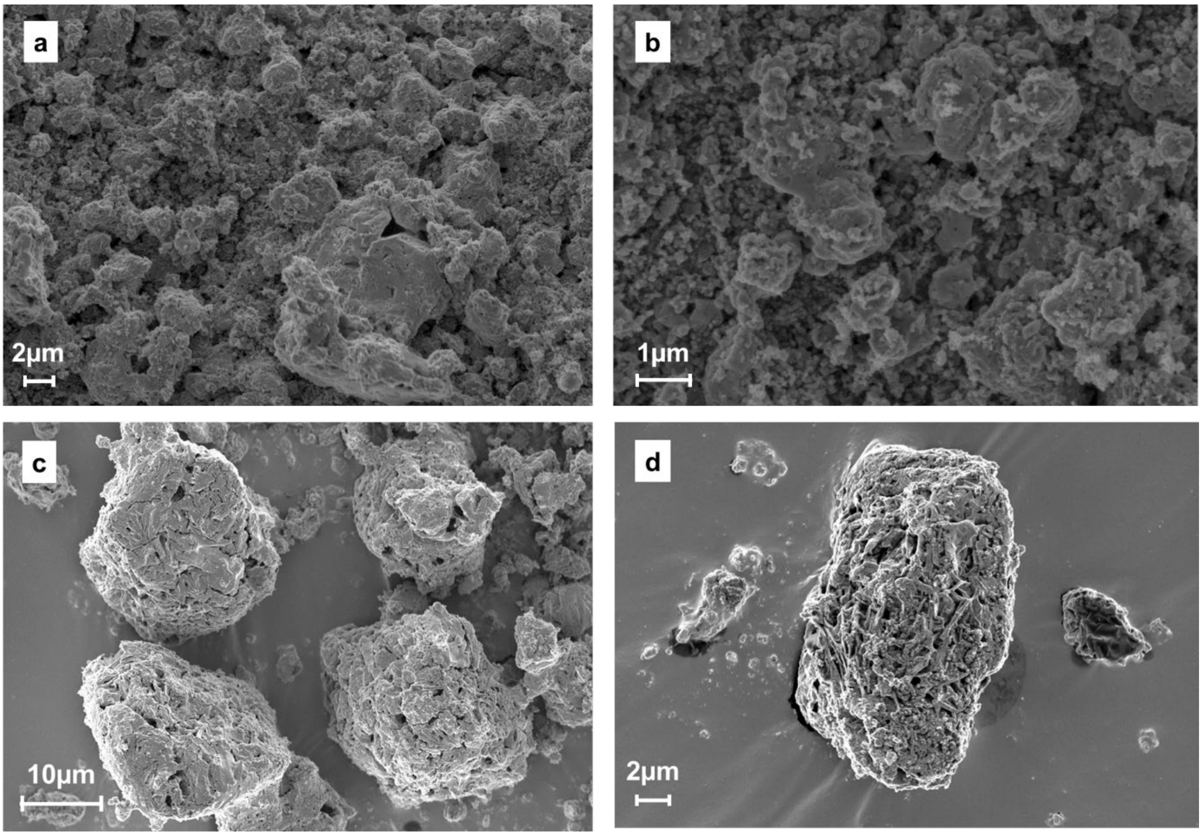

Figure S5. SEM images of 2:7:1 (TAQ:LPSCl:CB) cathode particles. **a, b,** Pristine (before cycling) and **c, d,** after 100 cycles at 25 mA g^{-1}.

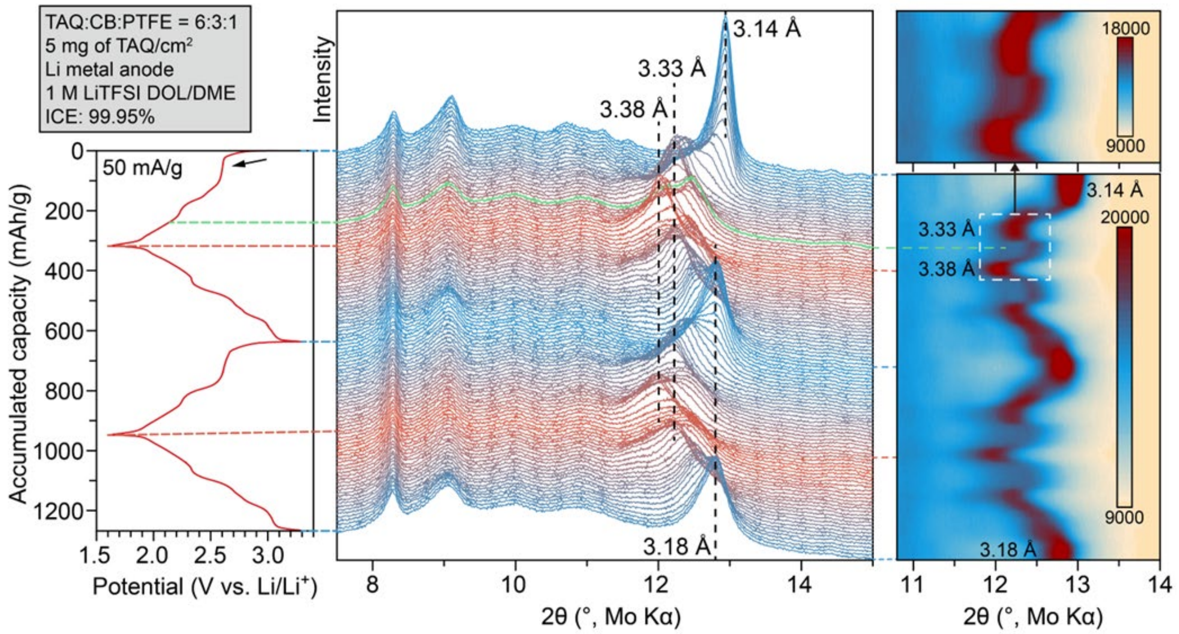

Figure S6. In situ PXRD patterns of a TAQ cathode in a liquid electrolyte system during electrochemical cycling. Reversible shifts in the characteristic d_{102} peak indicate interlayer spacing changes during lithiation and delithiation. The volumetric expansion of TAQ upon full lithiation, derived from the d_{102} spacing variation, is approximately 6.3%.

Table S1. Comparison of volumetric expansion for representative cathode materials upon lithiation

Material Type	Electrode Material	Volume Change (%)	Reference
Inorganic	LFP ($LiFePO_4$)	6.5 – 6.8	[5]
	NMC111 ($LiNi_{0.33}Mn_{0.33}Co_{0.33}O_2$)	3.4	[6], [7]
	NMC622 ($LiNi_{0.6}Mn_{0.2}Co_{0.2}O_2$)	5.2	
	NMC811 ($LiNi_{0.8}Mn_{0.1}Co_{0.1}O_2$)	7.8	
**Organic**	**TAQ (This work)**	**~ 6.3**	**-**

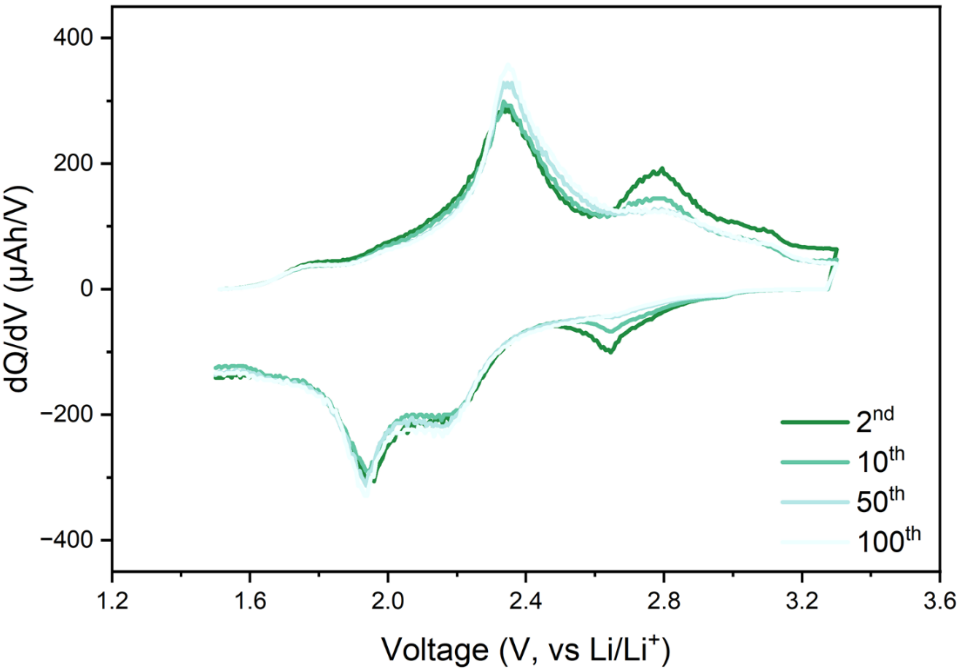

Figure S7. Differential capacity (*d*Q/*d*V) plots at selected cycles for the 2:7:1 (TAQ:LPSCl:CB) cathode cycled at 25 mA g^{-1}.

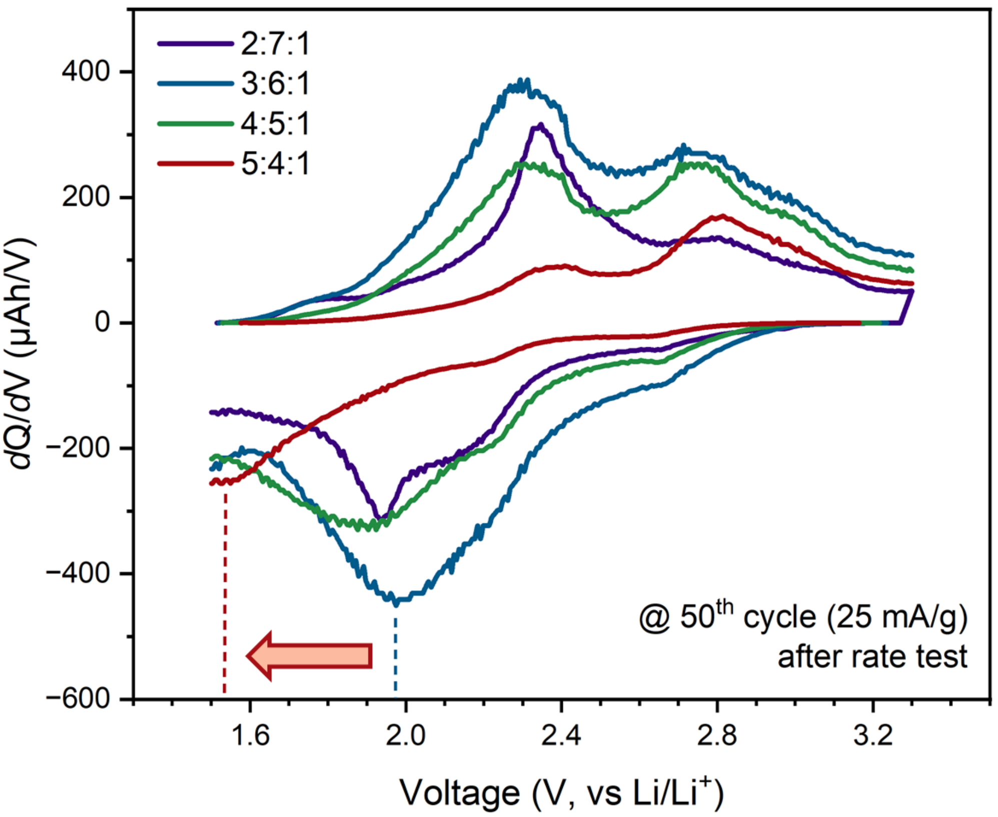

Figure S8. Differential capacity (dQ/dV) plots at the 50th cycle for TAQ cathodes with different compositions following rate capability testing. The arrow indicates a significant increase in discharge overpotential for the 50 wt% TAQ composition compared to others.

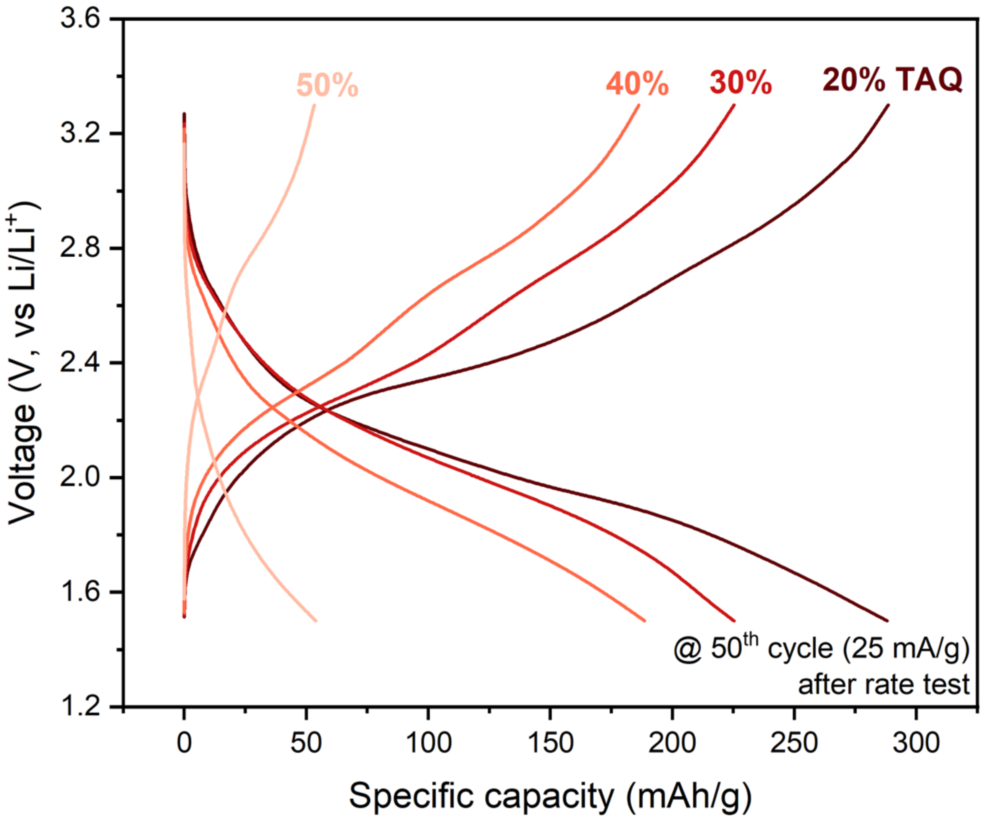

Figure S9. Galvanostatic charge-discharge (GCD) profiles at the 50th cycle for TAQ cathodes with different compositions following rate capability testing.

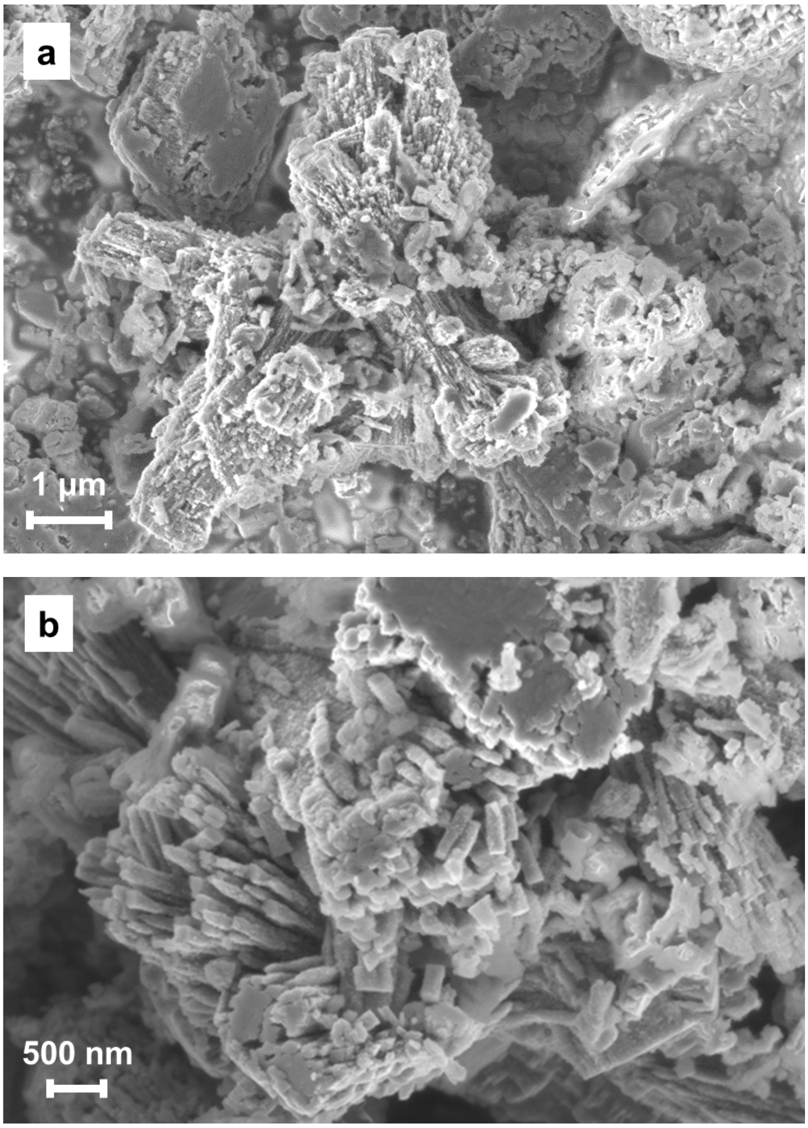

Figure S10. SEM images of as-synthesized low-crystallinity TAQ. **a,** Scale bar: 1 µm. **b,** Scale bar: 500 nm. The low-crystallinity sample exhibits smaller particle sizes (nanocrystalline) and indistinct crystal morphology compared to the high-crystallinity sample.

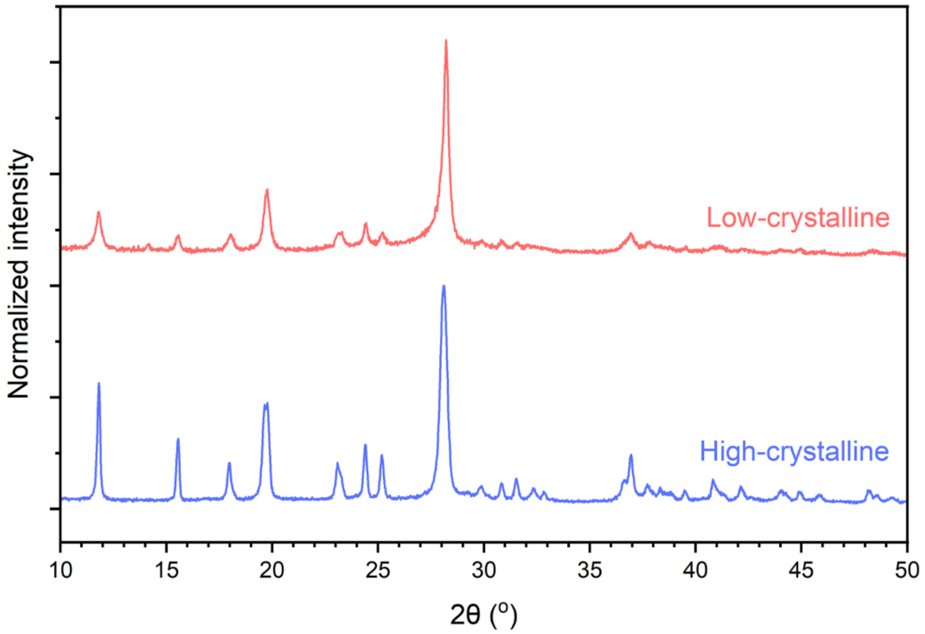

Figure S11. PXRD patterns of high- and low-crystallinity TAQ samples.

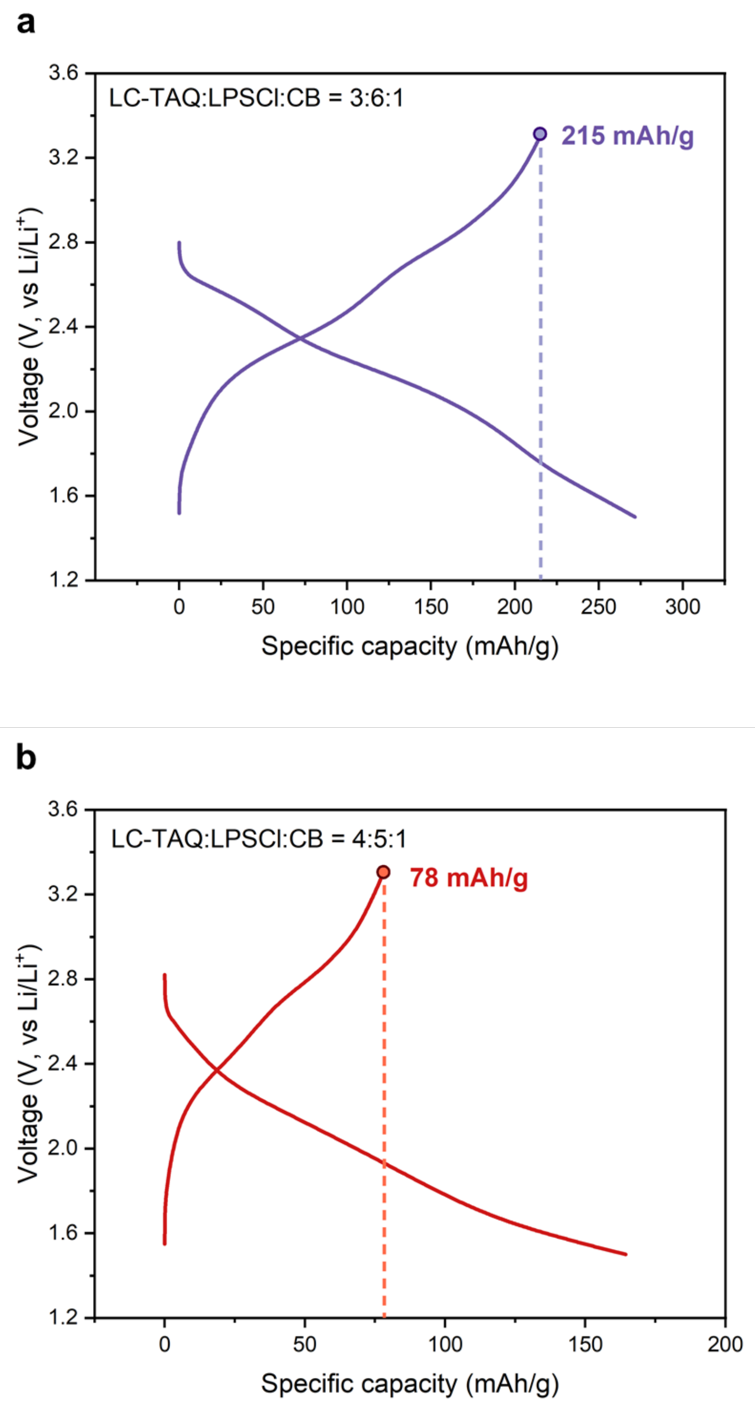

Figure S12. Galvanostatic charge-discharge (GCD) profiles of two representative cathode compositions with low-crystallinity TAQ (LC-TAQ) at the first cycle. **a,** 3:6:1 and **b,** 4:5:1 (LC-TAQ:LPSCl:CB) half cells with Li metal anodes (charge/discharge rate: 25 mA g^{-1}). Initial GCD profiles indicate specific capacities of 215 and 78 mAh g^{-1}, respectively, at the first charge.

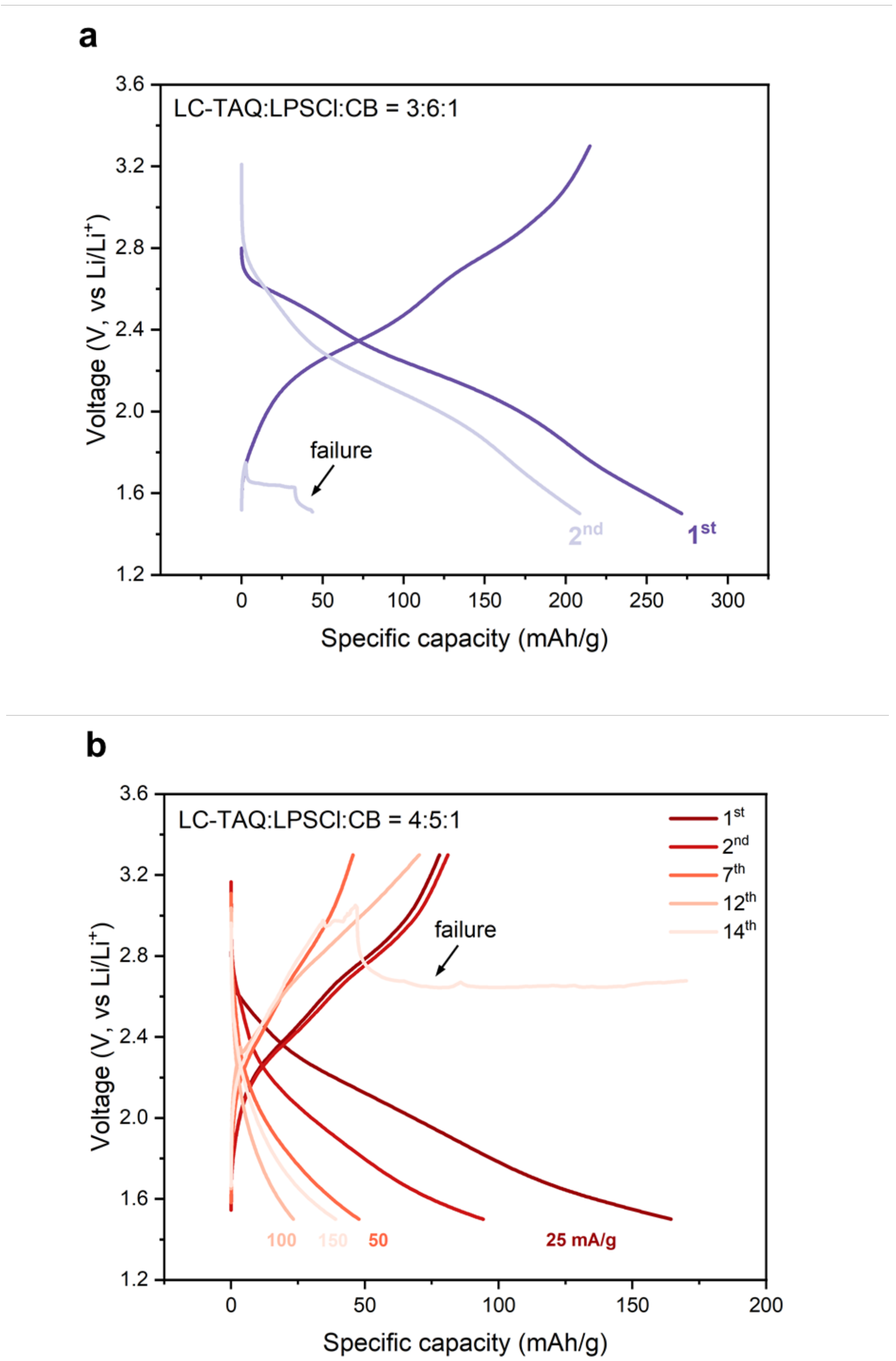

Figure S13. Galvanostatic charge-discharge (GCD) profiles at selected cycles for cathodes prepared with low-crystallinity TAQ (LC-TAQ). **a,** 3:6:1 (LC-TAQ:LPSCl:CB) cathode at 25 mA g^{-1} showing early-stage failure. **b,** 4:5:1 (LC-TAQ:LPSCl:CB) cathode during rate capability testing, demonstrating failure at high current density.

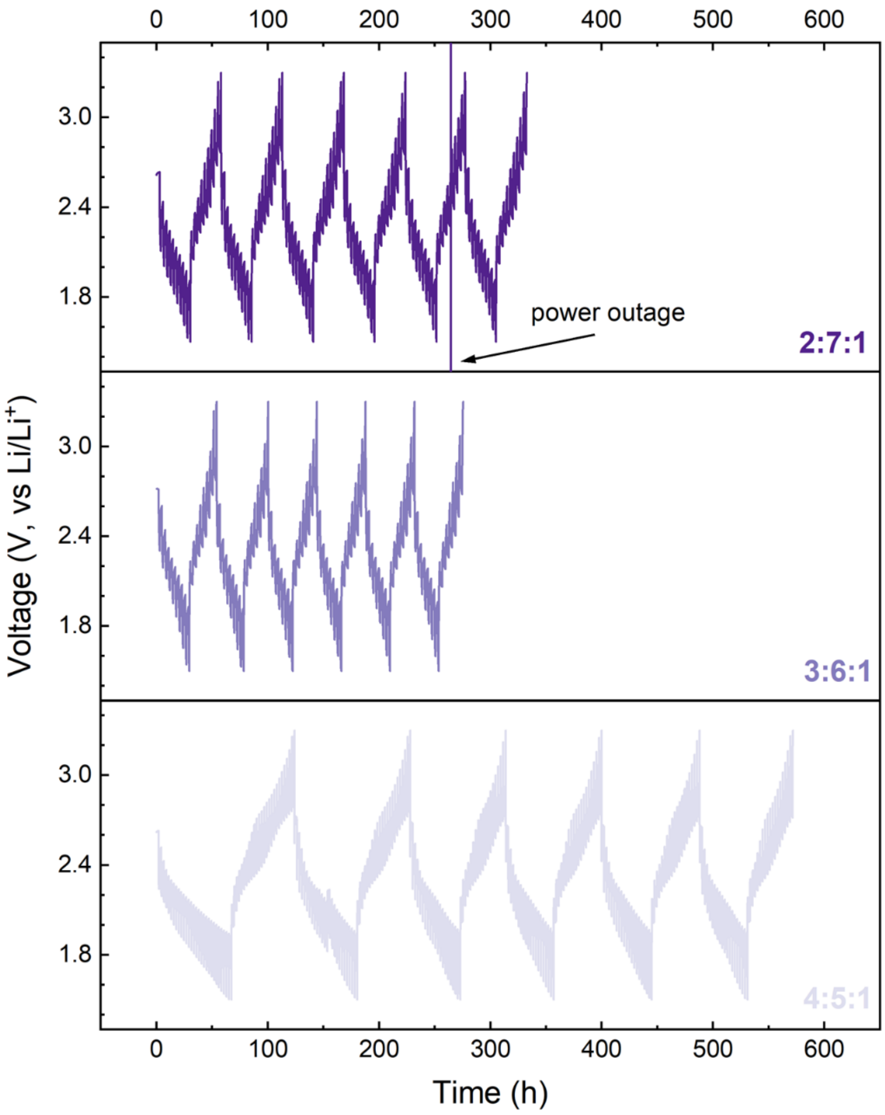

Figure S14. Raw GITT data for three cathode compositions with varying experimental conditions. The 2:7:1 and 3:6:1 cathodes were measured using 1-hr pulse at 25 mA g^{-1} followed by 2-hr rest periods, while the 4:5:1 cathode employed 15-min pulse at 25 mA g^{-1} with 2-hr rest periods. Voltage cutoffs were set at 1.5 V and 3.3 V vs Li/Li^{+}.

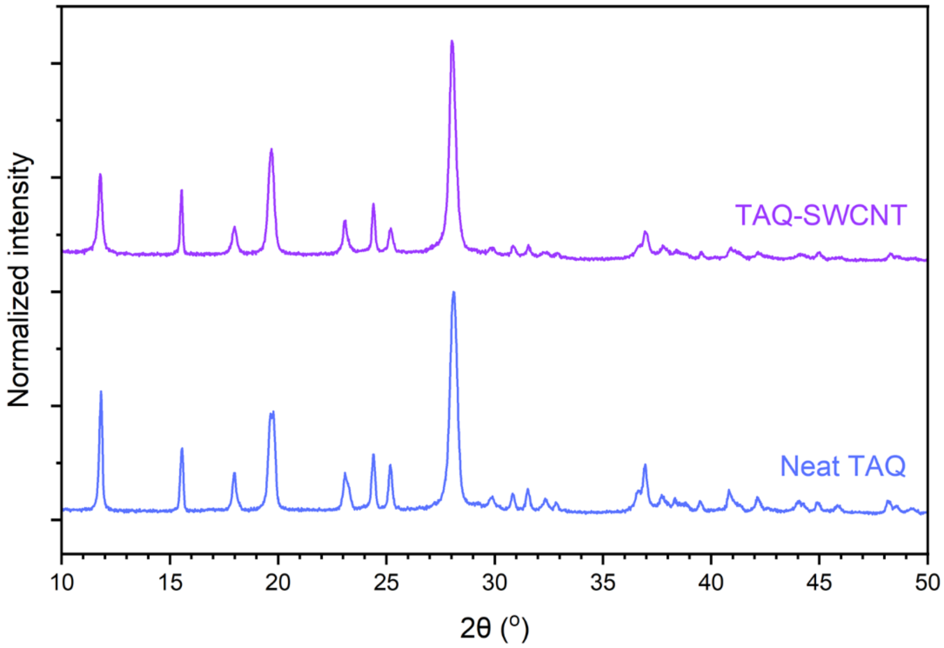

Figure S15. PXRD patterns of neat TAQ and TAQ-SWCNT composite samples.

Table S2. Elemental analysis of the TAQ-SWCNT composite

	C (%)	H (%)	N (%)
**Trial #1**	48.10	3.06	26.05
**Trial #2**	47.77	2.87	25.86

Detailed calculation and discussion for SWCNT content determination:

Assuming all nitrogen originates exclusively from TAQ (chemical formula $C_6N_6O_4H_{8+2n}$, $0 \leq n \leq 1$), the carbon contributions from TAQ can be calculated as 44.66 % and 44.33 % for the two analyzed samples, respectively, based on the stoichiometric ratios. Similarly, oxygen contributions from TAQ are calculated as 19.85% and 19.70%, respectively. Subtracting these TAQ-derived carbon contributions from the total carbon content yields SWCNT-derived carbon of 3.44% in both samples. Since the carboxyl-functionalized SWCNT contains 1.0-3.0 atom% carboxylic acid groups, hydrogen and oxygen contributions from these functional groups account for approximately 2.7 wt% of the SWCNT mass. Therefore, the TAQ-SWCNT composite material contains approximately 3.65 wt% SWCNT, calculated according to the equation below.

$$\text{Trial \#1:} \quad \frac{3.44 \times 1.027}{48.10 + 3.06 + 26.05 + 19.85} \times 100 = 3.64\ (\text{wt\%})$$

$$\text{Trial \#2:} \quad \frac{3.44 \times 1.027}{47.77 + 2.87 + 25.86 + 19.70} \times 100 = 3.67\ (\text{wt\%})$$

This experimentally determined value can be compared with the theoretical SWCNT content based on the synthetic procedure. In the synthesis, 1 mg of carboxyl-functionalized SWCNT and 100 mg of TABQ were used, yielding 22.5 mg of TAQ-SWCNT composite material. If all added SWCNT were incorporated into the composite, the theoretical SWCNT content would be 4.3 wt%. The lower experimental value (3.65 wt%) suggests that a portion of the SWCNT remained unreacted or was lost during synthesis and purification, rather than forming the composite with TAQ.

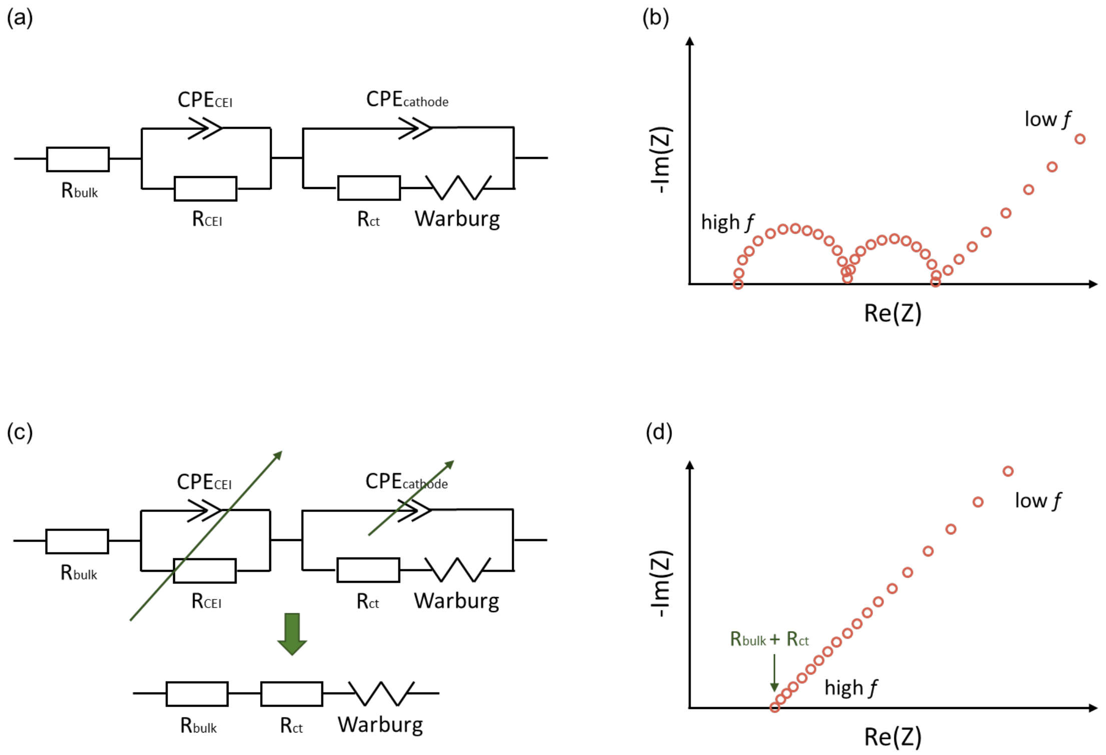

Figure S16. Equivalent circuit models and corresponding Nyquist plots for battery systems. **a,** Equivalent circuit model for a typical liquid electrolyte battery half-cell. **b,** Expected Nyquist plot showing two semicircles and Warburg tail. **c,** Simplified equivalent circuit model for TAQ ASSBs **d,** Expected Nyquist plot showing predominantly linear behavior.

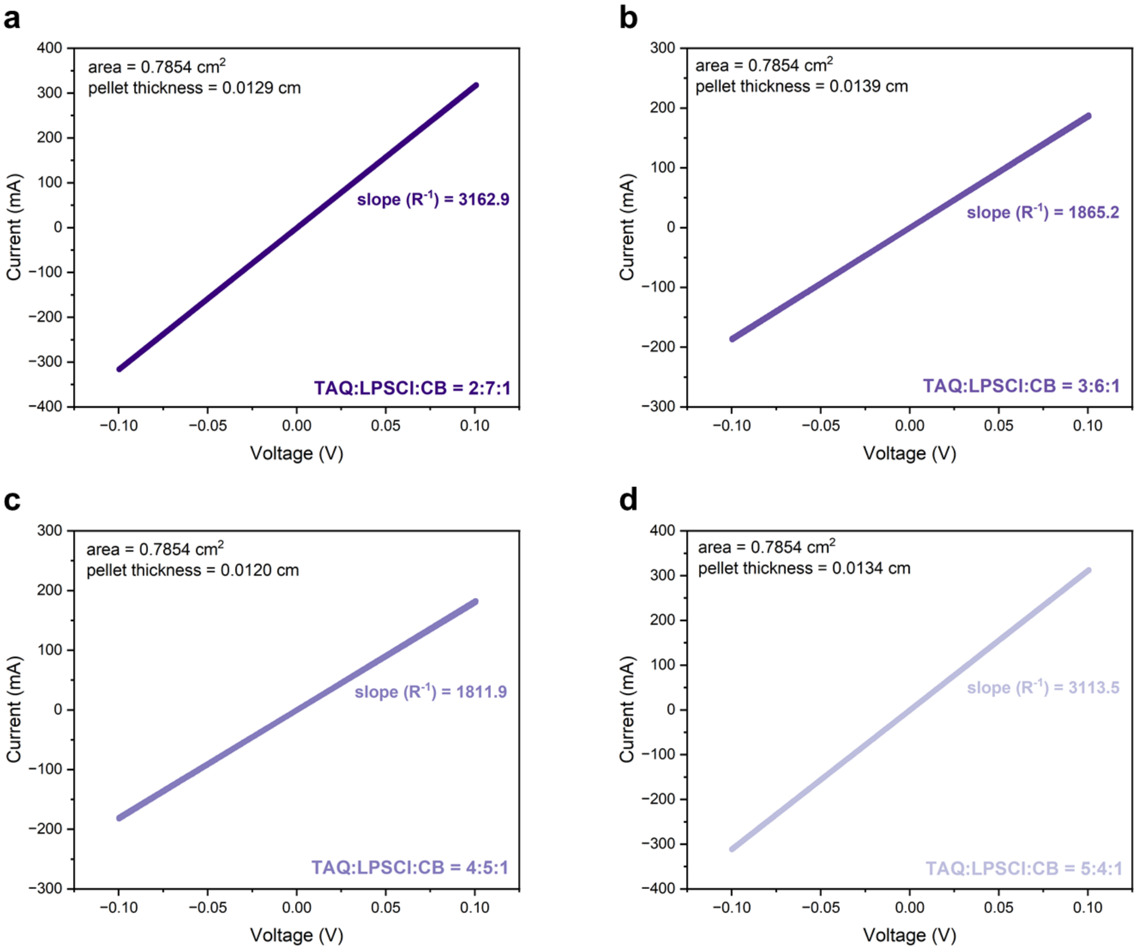

Figure S17. Two-probe electrical conductivity measurements of TAQ composite cathode pellets. Current–voltage (I–V) curves for **a,** 2:7:1, **b,** 3:6:1, **c,** 4:5:1, and **d,** 5:4:1 TAQ:LPSCl:CB cathodes.

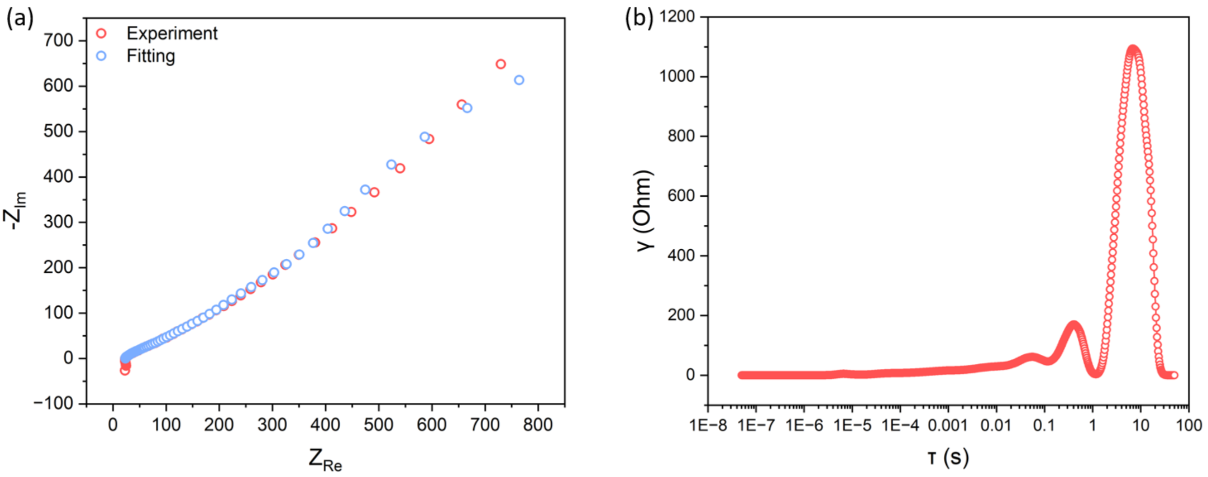

Figure S18. Distribution of relaxation times (DRT) analysis of representative 2:7:1 (TAQ:LPSCl:CB) composite cathode in the fully discharged state. **a,** Experimental Nyquist plot and corresponding fitting. **b,** Distribution of relaxation times derived from the fitted Nyquist plot. Dominant peaks at longer relaxation times (10^{-1}–10^{1} s) indicate mass-transfer limited processes, in contrast to charge-transfer processes that typically occur at shorter timescales.[8,9] The analysis has been conducted using available online software.[10] The regularization parameter λ has been set as 0.001.

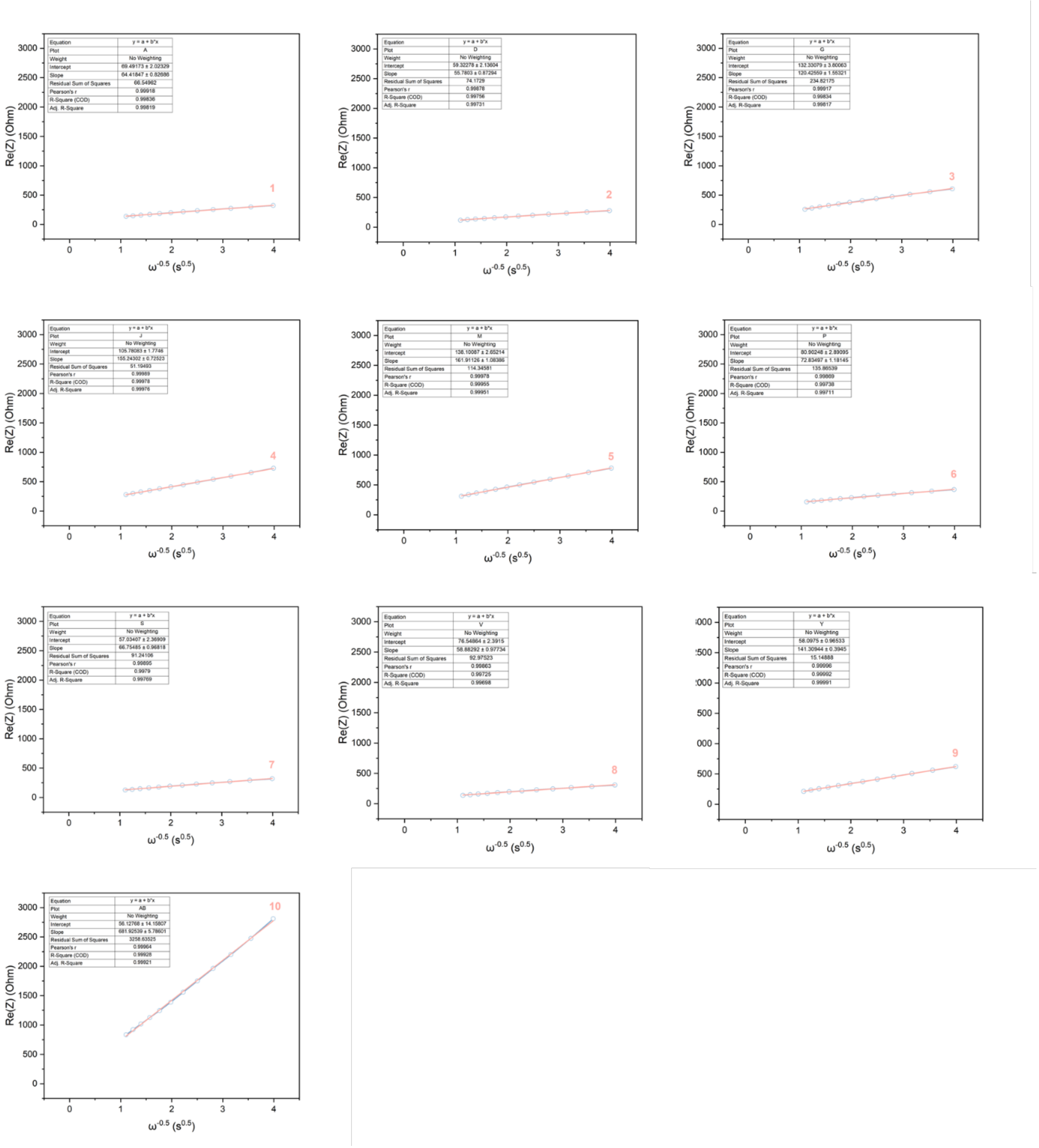

Figure S19. Warburg coefficient extraction for the 2:7:1 (TAQ:LPSCl:CB) cathode at different states of charge. Plots of $\omega^{-0.5}$ versus Re(Z) plots in the low-frequency regime (blue lines) with linear regression fits (red lines) at each voltage point.

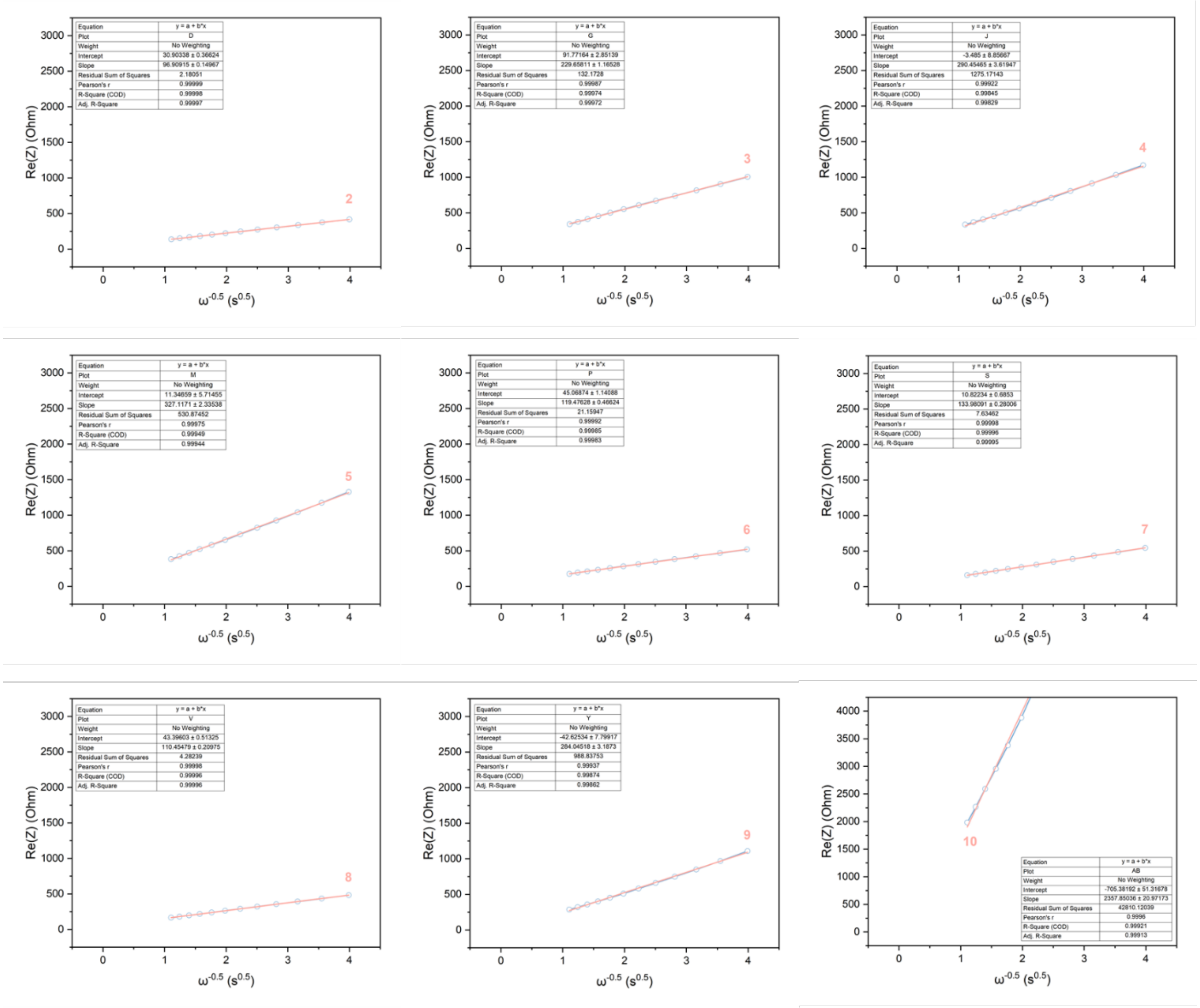

Figure S20. Warburg coefficient extraction for the 3:6:1 (TAQ:LPSCl:CB) cathode at different states of charge. Plots of $\omega^{-0.5}$ versus Re(Z) plots in the low-frequency regime (blue lines) with linear regression fits (red lines) at each voltage point.

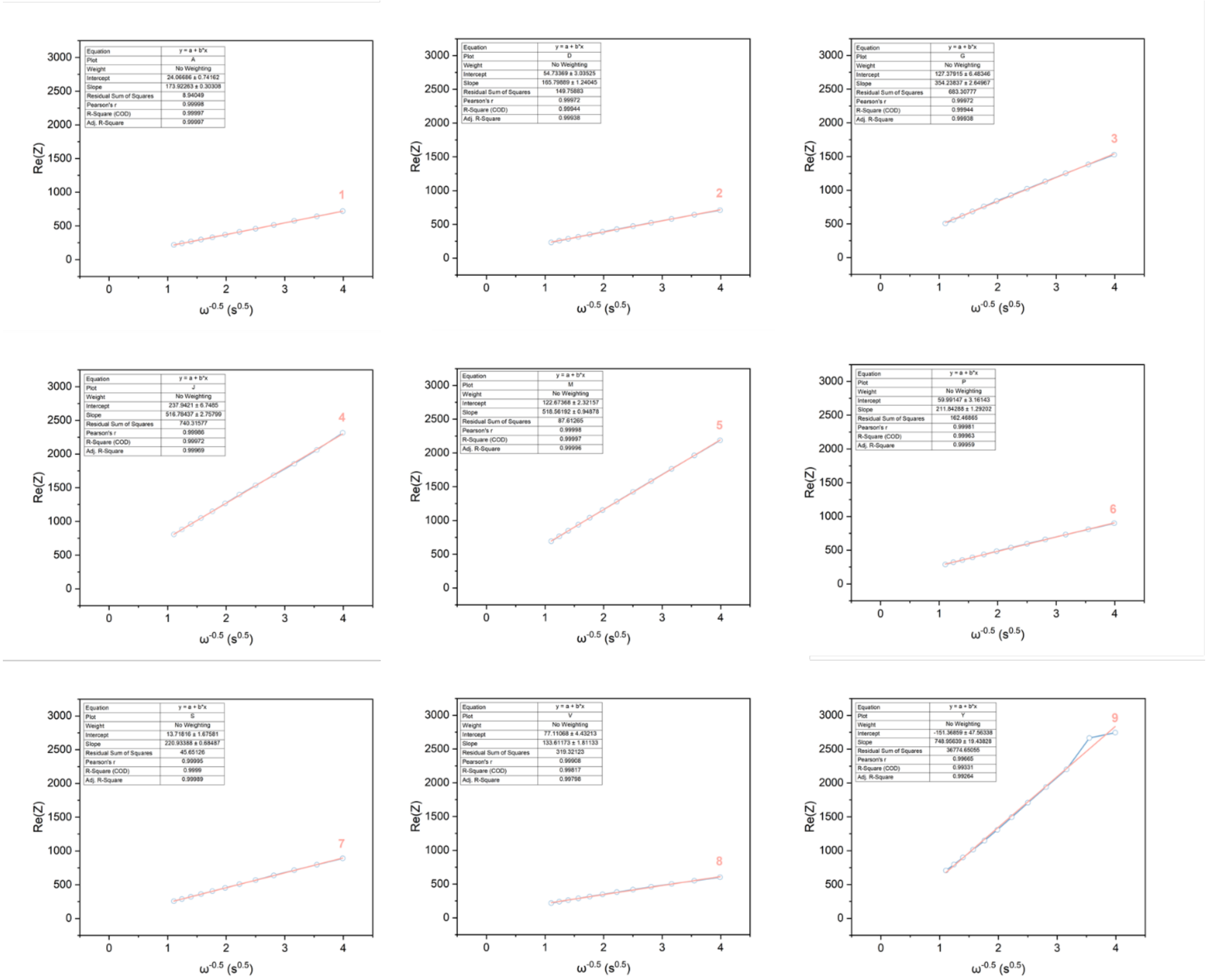

Figure S21. Warburg coefficient extraction for the 4:5:1 (TAQ:LPSCl:CB) cathode at different states of charge. Plots of $\omega^{-0.5}$ versus Re(Z) plots in the low-frequency regime (blue lines) with linear regression fits (red lines) at each voltage point.

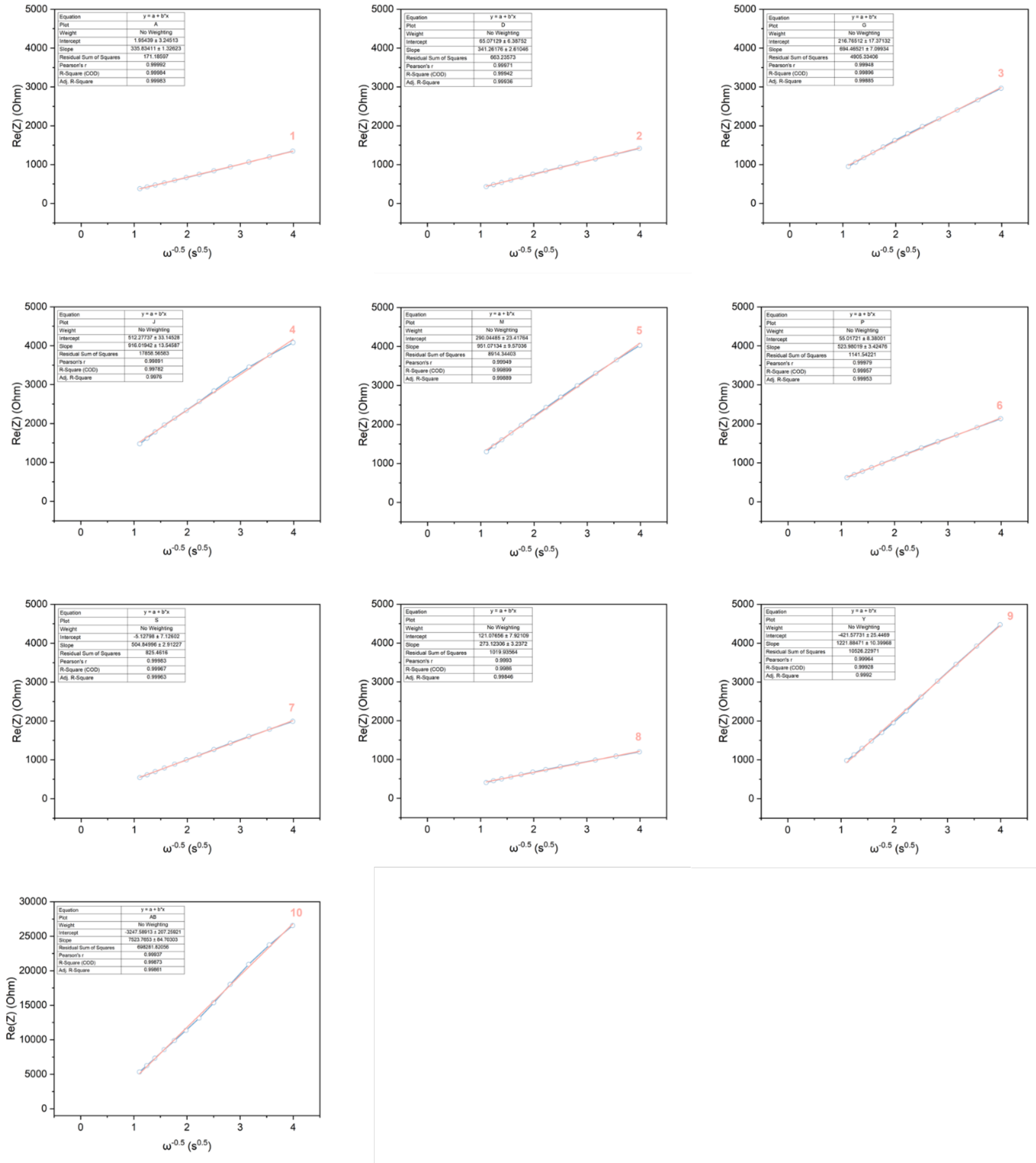

Figure S22. Warburg coefficient extraction for the 5:4:1 (TAQ:LPSCl:CB) cathode at different states of charge. Plots of $\omega^{-0.5}$ versus Re(Z) plots in the low-frequency regime (blue lines) with linear regression fits (red lines) at each voltage point.

Table S3. Performance comparison of representative cathode materials in LPSCl-based ASSBs

Material Type	Electrode Material	Active Material Content (%)	Specific Capacity (mAh/g)	Cycling Retention	Reference
Inorganic	LFP	Incompatible with LPSCl			[11]
	NMC*	70	~150	96.3% (50th cycle) @ 0.5 mA/cm^2	[12]
Organic	PTO	40	411	75% (100th cycle) @ 41 mA/g	[13]
	COF-TRO	50	270	96% (100th cycle) @ 27 mA/g	[14]
	PAQS-G	43	178	94% (200th cycle) @ 18 mA/g	[15]
	PI	50	190	105% (100th cycle) @ 18 mA/g	[16]
	**TAQ (This work)**	**20 - 50**	**310**	**96% (100th cycle) @ 25 mA/g**	**-**

*$LiNbO_3$-coated NMC was used as an active material.

References

1. Luo, Z.; Liu, L.; Ning, J.; Lei, K.; Lu, Y.; Li, F.; Chen, J. A Microporous Covalent–Organic Framework with Abundant Accessible Carbonyl Groups for Lithium-Ion Batteries. *Angewandte Chemie International Edition* **2018**, 57 (30), 9443–9446. https://doi.org/10.1002/anie.201805540.
2. Sun, L.; Park, S. S.; Sheberla, D.; Dincă, M. Measuring and Reporting Electrical Conductivity in Metal–Organic Frameworks: CD2(TTFTB) as a case study. *Journal of the American Chemical Society* **2016**, 138 (44), 14772–14782. https://doi.org/10.1021/jacs.6b09345.
3. Yang, X.; Hu, Y.; Dunlap, N.; Wang, X.; Huang, S.; Su, Z.; Sharma, S.; Jin, Y.; Huang, F.; Wang, X.; Lee, S.; Zhang, W. A Truxenone-based Covalent Organic Framework as an All-Solid-State Lithium-Ion Battery Cathode with High Capacity. *Angewandte Chemie International Edition* **2020**, 59 (46), 20385–20389. https://doi.org/10.1002/anie.202008619.
4. Haldar, S.; Roy, K.; Kushwaha, R.; Ogale, S.; Vaidhyanathan, R. Chemical exfoliation as a controlled route to enhance the anodic performance of COF in LIB. *Advanced Energy Materials* **2019**, 9 (48). https://doi.org/10.1002/aenm.201902428.
5. Koyama, Y.; Chin, T. E.; Rhyner, U.; Holman, R. K.; Hall, S. R.; Chiang, Y.-M. Harnessing the Actuation Potential of Solid-State Intercalation Compounds. *Advanced Functional Materials* **2006**, 16 (4), 492–498. https://doi.org/10.1002/adfm.200500633.
6. Choi, J.; Manthiram, A. Role of Chemical and Structural Stabilities on the Electrochemical Properties of Layered $LiNi_{1/3}Mn_{1/3}Co_{1/3}O_2$ Cathodes. *Journal of The Electrochemical Society* **2005**, 152 (9), A1714. https://doi.org/10.1149/1.1954927.
7. Biasi, L.; Kondrakov, A. O.; Geßwein, H.; Brezesinski, T.; Hartmann, P.; Janek, J. Between Scylla and Charybdis: Balancing among Structural Stability and Energy Density of Layered NCM Cathode Materials for Advanced Lithium-Ion Batteries. *The Journal of Physical Chemistry C* **2017**, 121 (47), 26163–26171. https://doi.org/10.1021/acs.jpcc.7b06363.
8. Semerukhin, D.Yu.; Kubarkov, A.V.; Sergeyev, V.G.; Semenikhin, O.A.; Antipov, E.V. Analysis of the Distribution of Relaxation Times (DRT) Responses of Li-Ion Cells as a Function of Their Preparation Conditions. *Electrochimica Acta* **2024**, 486, 144092. https://doi.org/10.1016/j.electacta.2024.144092.
9. Zhao, Y.; Kücher, S.; Jossen, A. Investigation of the Diffusion Phenomena in Lithium-Ion Batteries with Distribution of Relaxation Times. *Electrochimica Acta* **2022**, 432, 141174. https://doi.org/10.1016/j.electacta.2022.141174.
10. Wang, Z.; Wang, Y.; Py, B.; Maradesa, A.; Liu, J.; Wan, T. H.; Saccoccio, M.; Ciucci, F. DRTtools: Freely Accessible Distribution of Relaxation Times Analysis for Electrochemical Impedance Spectroscopy. *ACS Electrochemistry* **2025**, 1 (12), 2680-2689. https://doi.org/10.1021/acselectrochem.5c00334.
11. Cronk, A.; Chen, Y.-T.; Deysher, G.; Ham, S.-Y.; Yang, H.; Ridley, P.; Sayahpour, B.; Hoang, L.; An, J.; Jang, J.; Darren; Meng, Y. S. Overcoming the Interfacial Challenges of LiFePO4 in Inorganic All-Solid-State Batteries. *ACS Energy Letters* **2023**, 8 (1), 827–835. https://doi.org/10.1021/acsenergylett.2c02138.
12. Doerrer, C.; Capone, I.; Narayanan, S.; Liu, J.; Grovenor, C. R. M.; Pasta, M.; Grant, P. S. High Energy Density Single-Crystal NMC/Li_6PS_5Cl Cathodes for All-Solid-State Lithium-Metal Batteries. *ACS Applied Materials & Interfaces* **2021**, 13 (31), 37809–37815. https://doi.org/10.1021/acsami.1c07952.
13. Zhang, J.; Chen, Z.-Y.; Ai, Q.; Tanguy Terlier; Hao, F.; Liang, Y.; Guo, H.; Lou, J.; Yao, Y. Microstructure Engineering of Solid-State Composite Cathode via Solvent-Assisted Processing. *Joule* **2021**, 5 (7), 1845–1859. https://doi.org/10.1016/j.joule.2021.05.017.
14. Yang, X.; Hu, Y.; Dunlap, N.; Wang, X.; Huang, S.; Su, Z.; Sharma, S.; Jin, Y.; Huang, F.; Wang, X.; Lee, S.; Zhang, W. A Truxenone-Based Covalent Organic Framework as an All-Solid-State Lithium-Ion Battery Cathode with High Capacity. *Angewandte Chemie International Edition* **2020**, 59 (46), 20385–20389. https://doi.org/10.1002/anie.202008619.

15. Ji, W.; Zhang, X.; Xin, L.; Luedtke, A.; Zheng, D.; Huang, H.; Lambert, T.; Qu, D. A High-Performance Organic Cathode Customized for Sulfide-Based All-Solid-State Batteries. *Energy Storage Materials* **2022**, 45, 680–686. https://doi.org/10.1016/j.ensm.2021.12.015.
16. Ji, W.; Zhang, X.; Qu, H.; Xin, L.; Luedtke, A. T.; Huang, H.; Lambert, T. H.; Qu, D. Polyimide as a Durable Cathode for All-Solid-State Li (Na)−Organic Batteries with Boosted Cell-Level Energy Density. *Nano Energy* **2022**, 96, 107130. https://doi.org/10.1016/j.nanoen.2022.107130.
